# Catulin Based Reporter System to Track and Characterize the Population of Invasive Cancer Cells in the Head and Neck Squamous Cell Carcinoma

**DOI:** 10.3390/ijms23010140

**Published:** 2021-12-23

**Authors:** Kamila Karpińska, Mateusz Gielata, Aleksandra Gwiazdowska, Łukasz Boryń, Agnieszka Kobielak

**Affiliations:** 1 Laboratory of the Molecular Biology of Cancer, Centre of New Technologies, University of Warsaw, 00-927 Warsaw, Poland; k.karpinska@cent.uw.edu.pl (K.K.); m.gielata@cent.uw.edu.pl (M.G.); a.gwiazdowska@cent.uw.edu.pl (A.G.); 2 Laboratory of Stem Cells, Tissue Development and Regeneration, Centre of New Technologies, University of Warsaw, 00-927 Warsaw, Poland; l.boryn@cent.uw.edu.pl

**Keywords:** catulin, *CTNNAL1*, head and neck squamous cell carcinoma, HNSCC, invasion, EMT

## Abstract

Head and neck squamous cell carcinoma (HNSCC) is an aggressive tumor with a poor prognosis due to late diagnosis and loco-regional metastasis. Partial or more complete epithelial–mesenchymal transition (EMT) plays a role in tumor progression; however, it remains a challenge to observe the EMT in vivo, due to its transient nature. Here, we developed a novel catulin promoter-based reporter system that allows us to isolate and characterize in vivo a small fraction of invasive cancer cells. The analyses of tumors revealed that Catulin-green fluorescent protein (GFP)-positive cells were enriched in clusters of cells at the tumor invasion front. A functional genomic study unveiled genes involved in cellular movement and invasion providing a molecular profile of HNSCC invasive cells. This profile overlapped partially with the expression of signature genes related to the partial EMT available from the single cell analysis of human HNSCC specimens, highlighting the relevance of our data to the clinical disease progression state. Interestingly, we also observed upregulations of genes involved in axonal guidance—L1 cell adhesion molecule (*L1CAM*), neuropilin-1, semaphorins, and ephrins, indicating potential interactions of cancer cells and neuronal components of the stroma. Taken together, our data indicated that the catulin reporter system marked a population of invasive HNSCC cells with a molecular profile associated with cancer invasion.

## 1. Introduction

Head and neck carcinomas (HNC) are malignant neoplasms that affect important anatomical structures of the upper digestive tract and respiratory system, such as the tongue, mouth, pharynx, larynx, nasal cavity, sinuses, and salivary glands, and often affect key sensory nerves in the peripheral nervous system. The main risk factors associated with the development of this type of cancer are tobacco smoke [1], high-percentage alcohol [2], as well as infection with the human papilloma virus (HPV) [3]. Head and neck squamous cell carcinoma (HNSCC), which accounts for the majority of HNC cases, is the sixth most common cancer in the world, with a low and unchanged 50% 5-year survival rate [4,5]. This poor survival is likely due to the fact that local invasion, lymph node involvement, and metastasis are often present at the time of diagnosis [6].

An important aspect of HNSCC progression is the specific tumor microenvironment (TME), which consists of the extracellular matrix (ECM) as well as resident and recruited cells in the vicinity of cancer. These cells include cancer-associated fibroblasts (CAFs), which in the head and neck area are neural-crest-derived, adipose cells, immune-inflammatory cells as well as nerve, blood, and lymphatic vascular networks [7,8]. The progression and clinical outcome of cancer depends on complex interactions between tumor and stromal cells in the TME [9]. This cross-talk takes place through paracrine signaling or direct interactions between cells, which leads to bidirectional remodeling, enabling tumor cell proliferation, invasion, and migration followed by metastasis [10].

Metastasis is a complex multistep process that constitutes the main cause of death from many types of cancer [11]. During metastasis, cancer cells escape from the primary tumor, invade the surrounding tissues and enter the circulation [12]. Some cancer cells can survive in lymphatic or blood vessels and reach distal sites. Finally, the survivors extravasate and colonize in the second organ [13]. Apart from blood and lymphatic vessels, evidence indicates that neurogenesis (increased number of neurons) and axonogenesis (tumor-induced neural sprouting toward the TME) also play a vital role in tumorigenesis and metastasis. Cancer cells can also infiltrate inside or around nerves in the process called perineural invasion (PNI), which can be observed before lymphatic or vascular invasion [14,15]. High intratumoral nerve density correlates with poor prognosis and high recurrence across multiple solid tumor types [15]. Recent research has shown that cancer cells express neurotrophic markers such as nerve growth factor, brain-derived neurotrophic factor, and glial-cell-derived neurotrophic factor, and release axon-guidance molecules such as ephrin B1 to promote axonogenesis [16]. PNI in head and neck cancers is a significant cause of mortality, and it is one of the markers of poor prognosis for patients [17,18,19]. Prevalence rates of PNI in HNC ranges from 25% to 80%.

The epithelial–mesenchymal transition (EMT), the conversion of tumor cells from an epithelial to a mesenchymal phenotype, plays a key role in cancer metastasis [20]. During the EMT process, cancer cells exhibit the downregulation of E-cadherin and α-catenin, reduction of cell adhesion, and enhanced migration and invasiveness [21,22]. The process of the EMT is also closely linked with the acquisition of stem cell properties, including the expression levels of HNSCC CSC markers CD44, CD133, or ALDH1, which are associated with metastasis and treatment resistance. Therefore, a thorough understanding of the nature and the molecular basis of the EMT is of clinical importance to potentially increase the effectiveness of therapy and the survival of patients with HNSCC. However, due to the high plasticity and reversible nature of the EMT, the research of this process is very challenging [23]. The EMT can be context-dependent, occurring in distinct cellular populations at particular sites within the tumor. Therefore, their functional characteristics, tumor initiation potential, and gene expression profiles can be masked by non-metastatic and non-invasive cells. The detection and characterization of such transient and plastic cells in vivo during cancer progression is critical to assess the impact of the EMT on the pathogenesis of metastatic neoplasms. Reliable molecular markers that would allow detecting and sorting cells that dynamically undergo a partial or complete EMT in vivo are missing. Therefore, the development of the novel reporter systems that would allow detection of cells during transition is needed.

In our previously studied mouse model, the conditional loss of cell–cell junction protein α-catenin in the epithelium resulted in an increased cell proliferation and migration and squamous cell carcinoma (SCC) such as phenotype. Microarray analysis comparing mouse α-catenin cKO keratinocytes, which failed to form cell–cell junctions, and WT epithelial cells showed an upregulation of a new α-catenin homolog, α-catenin-like 1 (catulin) [24]. We showed that catulin is highly expressed at the invasion front of malignant hHNSCC and the upregulation of catulin expression correlates with the transition of tumor cells from an epithelial to mesenchymal morphology. The knockdown of catulin in hHNSCC cell lines dramatically decreases the migratory and invasive potential of those cells in vitro and the metastatic potential in xenotransplants in vivo, indicating an important role of this protein in the process of cancer metastasis [25]. Interestingly, the ablation of catulin during normal mouse development results in defects in neural tube closure due to aberrations in active RhoA distribution, actin-myosin dynamics, and tension at cell–cell adhesion, indicating the crucial role of this protein in cellular processes [26]. It has also been shown that catulin is highly expressed in malignant melanoma cells and is a key factor in tumor development, invasion, and metastasis due to the downregulation of E-cadherin and the upregulation of mesenchymal markers such as N-cadherin, Snail/Slug, and the matrix metalloproteinases 2 and 9 [27], as well as contributing to chemoresistance by the activation of NF-κB and AP-1 and the phosphorylation of ERK [28]. Catulin is also upregulated in highly invasive non-small cell lung cancer (NSCLC) cell lines, which promotes cancer cell migration, invasion, and metastasis through activating the integrin-linked kinase (ILK)-mediated Akt-NF-κB signaling [29]. Since the expression and function of catulin correlate with the metastatic potential of SCC, we have developed a novel catulin promoter-based reporter system with a green fluorescent protein (Catulin-GFP) in stable SCC cell lines to mark and isolate in vivo a small invasive population of cancer cells. Using this approach, we were able to characterize molecular profiles and signaling pathways associated with cancer invasion that can be associated with the EMT process and the progression of HNSCC.

## 2. Results

### 2.1. Development of a Novel Catulin-Promoter-Based Reporter System to Label and Track the Population of Invasive Cancer Cells of HNSCC

**Figure 1:**
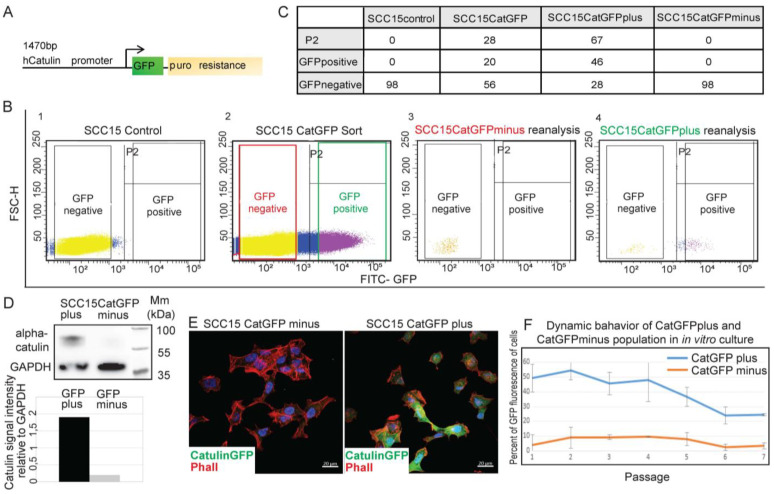
Catulin-promoter-based reporter system to label and track the population of invasive cancer cells of head and neck squamous cell carcinoma (HNSCC). (**A**) Schematic visualization of the reporter system. To generate the alpha-catulin reporter system, a human alpha-catulin promoter clone (containing a 1.5 kb insert corresponding to the 5’-flanking sequence from approximately 1400 upstream to 41 downstream of the human alpha-catulin gene transcription start site (TSS)) was placed upstream of a green fluorescent protein (GFP) in a 4400 bp vector with puromycin as a stable selection marker. The nucleotide identity and direction of the insert were verified by sequencing of both strands. (**B**) Fluorescence-activated cell sorting (FACS) analysis result of SCC15CatGFP cells in vitro. The fluorescence analysis results of the control SCC15 cells (**1**) and the sort of the reporter cell line SCC15CatGFP (**2**) are shown. SCC15CatGFP was sorted into two populations, i.e., GFP minus (cells with no expression of alpha-catulin, with no fluorescence of GFP) and GFP plus (cells with the expression of alpha-catulin and with fluorescence of GFP). P2 gate contained all the cells that had GFP fluorescence; however, the gate for sorting was set more restrictively, and only cells from the GFP-positive gate were sorted (green frame). The reanalysis of the sorted SCC15CatGFP reporter cell line (SCC15CatGFP minus (**3**) and SCC15CatGFP plus (**4**) populations) are shown. (**C**) Percentage quantification of each cell type (GFP-positive and GFP-negative) in each cell line (SCC15 Control, SCC15CatGFP, SCC15CatGFP plus, and SCC15CatGFP minus). (**D**) Western blot analysis of alpha-catulin protein levels in the sorted SCC15CatGFP plus and SCC15CatGFP minus cells. GAPDH protein was used as a loading control. The graph showed the catulin signal intensity calculated relative to that of GAPDH in ImageJ software. (**E**) Immunofluorescence of the sorted SCC15CatGFP plus and SCC15CatGFP minus cells seeded on coverslip glasses. Catulin-GFP immunofluorescence is indicated in green, phalloidin (actin) immunofluorescence is indicated in red, and 4′6-diamidino-2-phenylindole (DAPI) immunofluorescence is indicated in blue. Scale bar: 20 μm. (**F**) Dynamic behavior of the fluorescence signals of the SCC15CatGFP plus and SCC15CatGFP minus populations in an in vitro culture in passages from 1 to 7. The graph represents the average percentage of GFP-positive cells from three independent experiments. Standard error bars are shown.

We showed previously that the upregulation of catulin expression in vitro correlates with the transition of cancer cells from an epithelial to mesenchymal morphology, and the increased expression levels of EMT markers Vimentin and Snail [25]. To track and isolate invasive cancer cells of HNSCC, we took advantage of the specific expression and function of catulin, and developed a novel reporter system, where GFP expression was driven directly from the catulin short promoter (Figure 1A). After the stable transfection of SCC15 and SCC351 cell lines with the catulin reporter plasmid, followed by puromycin selection, the GFP fluorescence signals of established SCC15CatGFP and SCC351CatGFP cell lines were analyzed using flow cytometry (Figure 1B, Supplementary Figure S2A,A′). The corresponding SCC cell lines were used as a control, to set up two gates, i.e., GFP-negative and GFP-positive (gate P2, which cut off 99.9% of the SCC15 cells; Figure 1B(1), Supplementary Figure S2A(1)). The fluorescence analysis of the SCC15CatGFP cell line showed 28% of GFP-positive cells (gate P2; Figure 1B(2),C). Even more stringent conditions were used to sort the SCC15CatGFP and SCC351CatGFP cell lines into two populations for further analysis: Representative results for SCC15CatGFP plus (green frame which contained 20% of positive cells) and SCC15CatGFP minus (which contained 56% of negative cells) are shown in Figure 1B(2),C. To verify the efficiency of cell sorting, we reanalyzed the sorted SCC15CatGFP plus and SCC15CatGFP minus populations (Figure 1B(3,4)). As expected, in the SCC15CatGFP minus population, 98% of cells were GFP-negative (Figure 1B(3),C). In the SCC15CatGFP plus population, 67% of cells showed GFP fluorescence (P2 gate; Figure 1B(4),C), which indicated a significant enrichment in GFP-positive cells as compared to 20% of GFP positive cells in the starting population. The appearance of 28% of cells with no GFP fluorescence in the reanalyzed SCC15CatGFP plus population might be due to photo bleaching during the initial sort. To verify the system, the sorted SCC15CatGFP plus and SCC15CatGFP minus cells were seeded on coverslip glasses. After staining with phalloidin to visualize actin cytoskeleton and 4′6-diamidino-2-phenylindole (DAPI) to visualize nuclei, we analyzed fluorescence under a microscope. As expected, in cells sorted as the SCC15CatGFP plus, the majority were GFP-positive, in contrast to the SCC15CatGFP minus population, where there was no cell with GFP fluorescence (Figure 1E). To test the correlation of GFP fluorescence with the catulin protein level, we performed Western blot analysis of catulin levels in the sorted SCC15CatGFP plus and SCC15CatGFP minus populations (Figure 1D). This analysis confirmed a significant enrichment of catulin protein in the SCC15CatGFP plus population. We also tested the dynamics of the cells’ fluorescence in the SCC15CatGFP plus and SCC15CatGFP minus populations in an in vitro culture using an ArthurTM Fluorescence Cell Counter. GFP fluorescence was measured in every passage from the first to the seventh (Figure 1F). The first passage of the SCC15CatGFP plus population contained 50% of GFP-positive cells, and this number dropped with every passage stabilizing at the sixth passage, at the level of 25% of GFP-positive cells. This result was similar to the percentage of GFP-positive cells in the starting population of the established SCC15CatGFP cell line that was used for cell sorting (28%; Figure 1B(2), gate P2). In the SCC15CatGFP minus population, the fluctuation of GFP fluorescence was smaller and oscillated between 3% and 10% of GFP-positive cells, gaining stabilization at the sixth passage, with 3% of GFP-positive cells. These data collectively suggested that the Catulin-GFP reporter marks a specific population of SCC cells.

### 2.2. Catulin-GFP Reporter System Labels Cells with Higher Migratory, Invasive, and Tumorigenic Potential In Vitro

**Figure 2:**
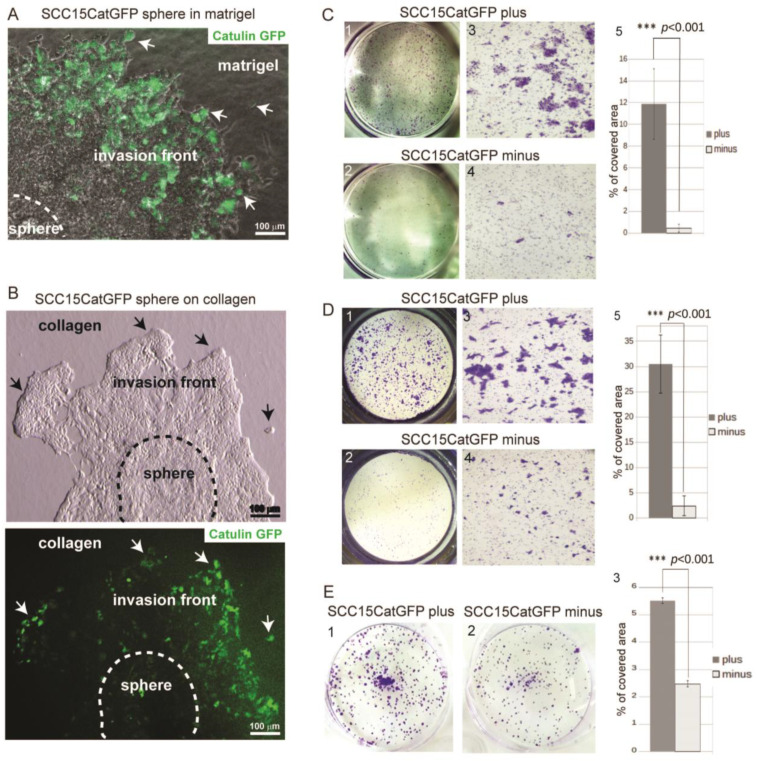
Migratory, invasive, and tumorigenic potential of the cells labeled with Catulin-GFP reporter system in vitro. (**A**) Sphere formed by SCC15CatGFP cells invading into Matrigel presented in a bright field and GFP fluorescence. Arrows indicate cells with the highest alpha-catulin (GFP fluorescence) expression at the invasion front. Scale bar: 100 µm. (**B**) Sphere formed by SCC15CatGFP cells placed on collagen presented in a bright field (**upper picture**) and GFP fluorescence (**lower picture**). Arrows indicate cells with the highest alpha-catulin (GFP fluorescence) expression at the invasion front. Scale bar: 100 µm. (**C**) Boyden chamber migration assays of the SCC15CatGFP plus and SCC15CatGFP minus populations. (**1**,**2**) Image of the entire insert. (**3**,**4**) Image of a randomly selected area at a higher magnification (4×). Cells were stained with crystal violet. (**5**) Quantification of the cells from both populations that migrated through the insert. *** statistical significance was assessed by t-test (*p* < 0.001). Standard error bars are shown. (**D**) Boyden chamber Matrigel invasion assays of the SCC15CatGFP plus and SCC15CatGFP minus populations. (**1**,**2**) Image of the entire insert. (**3**,**4**) Image of a randomly selected area at a higher magnification (4×). Cells were stained with crystal violet. (**5**) Quantification of cells from both population that invaded through the insert covered with Matrigel. *** statistical significance was assessed by t-test (*p* < 0.001). Standard error bars are shown. (**E**) Colony formation assays of the SCC15CatGFP plus (**1**) and SCC15CatGFP minus (**2**) populations. An equal number of cells of both populations were plated in duplicates into six-well plates. Cells were stained with crystal violet. (**3**) Quantification of colonies formed by both populations presented as a percent of the covered plate area. *** statistical significance was assessed by *t*-test (*p* < 0.001). Standard error bars are shown.

To investigate the invasive potential of GFP-positive cells in a three-dimensional (3D) model, the SCC15CatGFP reporter cell line was used to generate spheres that were then transferred into plates covered with Matrigel or collagen to observe cells invasion (Figure 2A,B). After three days, GFP-expressing cells were observed predominantly at the invasion front of the spheres, where the invading cells contacted with the Matrigel or collagen (arrows in Figure 2A,B). To compare the migratory and invasive potential of SCCCatGFP plus versus SCC15CatGFP minus cells, we performed Boyden chamber migration and Matrigel invasion assays of the sorted SCC15CatGFP plus and SCC15CatGFP minus populations (Figure 2C(1–5),D(1–5)). Both assays indicated increased migratory and invasive potential of the SCC15CatGFP plus population. We also analyzed the colony-forming capacities of the sorted SCC15CatGFP plus and SCC15CatGFP minus populations (Figure 2E(1–3)). The colony formation assays revealed that the SCC15CatGFP plus population was able to form more colonies in comparison to the SCC15CatGFP minus population. Taken together, obtained results indicated that the created SCC15CatGFP reporter system marked the population of cells with higher migratory, invasive, and tumorigenic potential in vitro.

### 2.3. Catulin-GFP Reporter System Marks a Small Population of Tumor Cells at the Invasive Front in a Xenograft Model of HNSCC That Looses Epithelial Marker E-Cadherin, Indicative of a Partial EMT

**Figure 3:**
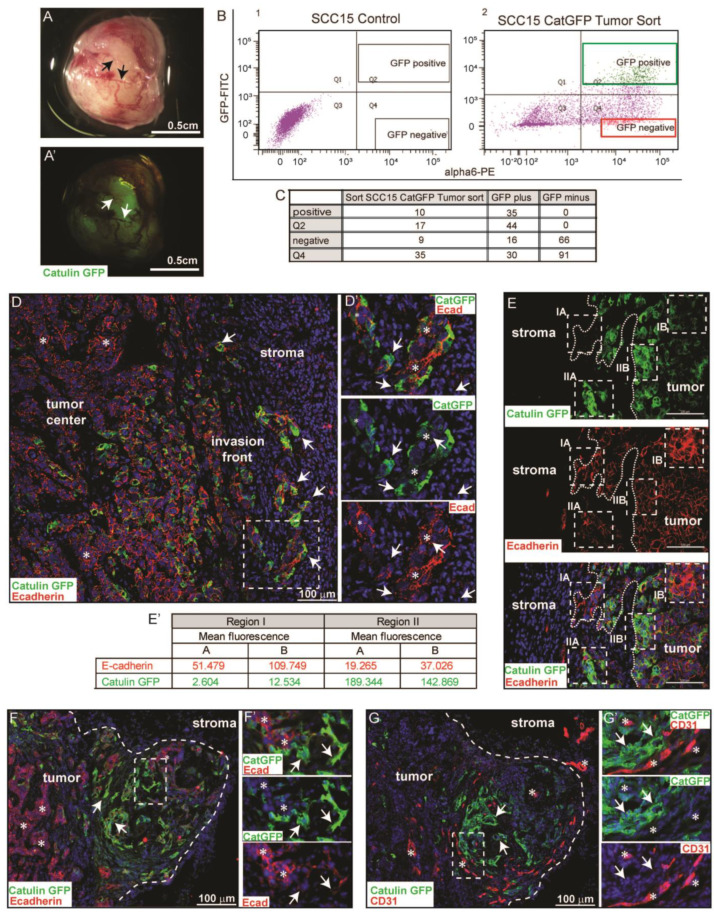
Catulin-GFP reporter system marked a small population of cells at the tumor invasive front. (**A**,**A**′) Dissection-scope picture of tumor formed after the injection of the SCC15CatGFP reporter cell line in a bright field (**A**) and GFP fluorescence (**A**′). Scale bar: 0.5 cm. (**B1**) Fluorescence analysis result of the control SCC15 cells. (**B2**) Fluorescence analysis result of the sorted SCC15CatGFP cells isolated from formed tumor. Alpha6-PE was used as an epithelial marker. Cells were FACS-sorted into two alpha6-PE-positive populations, i.e., SCC15CatGFP plus (GFP-positive; green frame) and SCC15CatGFP minus (GFP-negative; red frame). (**C**) Percentage quantification of each cell type (GFP-positive and GFP-negative) isolated from tumor SCC15CatGFP cells used for FACS and in each sorted cell population (SCC15CatGFP plus and SCC15CatGFP minus). In Q2 and Q4, gates were cells that had (Q2) or did not have (Q4) GFP fluorescence, respectively. However, gates for cell sorting were set more restrictively, and only cells from GFP-positive and GFP-negative gates were sorted (green and red frames). (**D**) Fluorescence analysis results of the section of tumor formed by SCC15CatGFP immunostained with the E-cadherin antibody. Tumor cells invading the stroma are indicated by arrows. Cells expressing high E-cadherin and low alpha-catulin levels are indicated with asterisks. GFP (alpha-catulin) fluorescence is indicated in green, E-cadherin fluorescence is indicated in red, and DAPI (nucleus) fluorescence is indicated in blue. Scale bar: 100 µm. The boxed area in (**D**) is enlarged in (**D**′). (**E**) Fluorescence analysis result of the section of tumor formed by SCC15CatGFP immunostained with the E-cadherin antibody. GFP (alpha-catulin) fluorescence is indicated in green, E-cadherin fluorescence is indicated in red, and DAPI (nucleus) fluorescence is indicated in blue. The border between the tumor and the stroma is indicated by the dashed line. Boxed areas are regions with strong E-cadherin (IA and IB) or GFP (IIA and IIB) fluorescence signals. Scale bar: 100 µm. (**E**′) Quantification of the mean fluorescence of E-cadherin and alpha-catulin (GFP) from regions I (A,B) and II (A,B). (**F**) Fluorescence analysis result of section of tumor formed by SCC15CatGFP immunostained with the E-cadherin antibody. Tumor cells expressing high alpha-catulin levels are indicated by arrows. Cells expressing high E-cadherin and low alpha-catulin levels are indicated by asterisks. GFP (alpha-catulin) fluorescence is indicated in green, E-cadherin fluorescence is indicated in red, and DAPI (nucleus) fluorescence is indicated in blue. Scale bar: 100 µm. The boxed area in (**F**) is enlarged in (**F**′). (**G**) Fluorescence analysis result of the section of tumor formed by SCC15CatGFP immunostained with the CD31 antibody. Tumor cells expressing high alpha-catulin levels are indicated by arrows. Cells expressing high CD31 levels are indicated by asterisks. GFP (alpha-catulin) fluorescence is indicated in green, CD31 fluorescence is indicated in red, and DAPI (nucleus) fluorescence is indicated in blue. Scale bar: 100 µm. The boxed area in (**G**) is enlarged in (**G**′). Arrows indicate areas enriched with GFP signal.

To study the nature and behavior of the labeled SCCCatGFP plus cells in vivo, we injected SCC15CatGFP and SCC351CatGFP reporter cell lines subcutaneously, into the neck area of NOD SCID mice. After formation, tumors were isolated and analyzed under the dissection scope in a bright field and with fluorescence (Figure 3A,A′). The tumors showed varying fluorescence with some areas enriched with GFP signals and vasculature (arrows in Figure 3A,A′). In order to characterize the catulin GFP+ cells at the transcriptional level, we isolated and performed fluorescence-activated cell sorting (FACS) of the SCC15CatGFP+/alpha6+ and SCC15CatGFP−/alpha6+ cells from tumors formed after the injection of the SCC15catulinGFP reporter cell line (Figure 3B). Integrin alpha6 was used as an additional marker for epithelial cancer cells. We set up the gates in a very stringent way, in which SCC15CatGFP+/alpha6+-positive cells accounted for 10% of the total SCC15CatGFP population (green gate) whereas the SCC15CatGFP−/alpha6+-negative population accounted for 9% of the total SCC15CatGFP population (red gate) (Figure 3C). The sorted cells were subjected to the reanalysis to confirm the enrichment in GFP within the GFP-positive fraction (GFP plus fraction in Figure 3C, Supplementary Figure S1D) and the lack of GFP-positive cells within the negative fraction (GFP minus fraction in Figure 3C, Supplementary Figure S1D). The same approach was applied to tumors derived from the SCC351CatGFP cell line (Supplementary Figure S2B–D). These analyses confirmed a proper cell sorting strategy, as we observed an enrichment of GFP-positive cells in the Catulin-GFP plus population and no GFP-positive cells in the SCC15CatGFP minus population. The presence of GFP-negative cells within the reanalyzed SCC15CCatGFP plus population might be due to laser photobleaching, similarly to the in vitro experiment. The fluorescence analysis for DAPI, indicating viability for all abovementioned cell populations, is presented in Supplementary Figure S1B,C. Compensation controls for cell sorting and analysis are presented in Supplementary Figure S1A. Small parts of the isolated tumors were fixed in PFA and embedded in an OCT compound, and sections were immunostained with epithelial marker E-cadherin and analyzed under a confocal microscope. Strong GFP fluorescence, indicating catulin-GFP reporter expression, was observed specifically at the tumor invasion front in clusters of cells invading the surrounding stroma (arrows in Figure 3D,D′). The further confocal microscopy analysis revealed that cells with high expression of the catulin reporter (strong GFP fluorescence) (boxed areas II in Figure 3D,E and arrows in Figure 3F,F′) showed a decreased E-cadherin expression, indicative of a partial EMT. On the contrary, in the tumor center, minimal GFP fluorescence correlated with a strong expression level of E-cadherin at the cell–cell contacts (asterisks in Figure 3D,D′ and asterisks in Figure 3F,F′). The mean fluorescence of GFP and E-cadherin from boxed areas in Figure 3E was quantified and is presented in the table in Figure 3E′. Since tumor areas with a strong GFP expression level was visibly enriched in vasculature (Figure 3A,A′). Tumor sections were also immunostained with endothelial marker CD31 antibody, which marked vasculature. Remarkably, an area with a newly formed tumor bud-like structure invading into the stroma was characterized by strong GFP expression, a decrease in cell–cell junctional E-cadherin staining (arrows in Figure 3F,G,G′), and an enrichment in a newly formed vasculature in the vicinity of the GFP-positive cells (asterisks in Figure 3F,G,G′). These observations validated the use of the Catulin-GFP reporter system to analyze the transcriptional profile of Catulin-GFP-positive cells as a population of tumor invasive cells.

### 2.4. Identification of Signature Genes of Invasive Catulin-GFP Reporter-Labeled Cancer Cells

**Figure 4:**
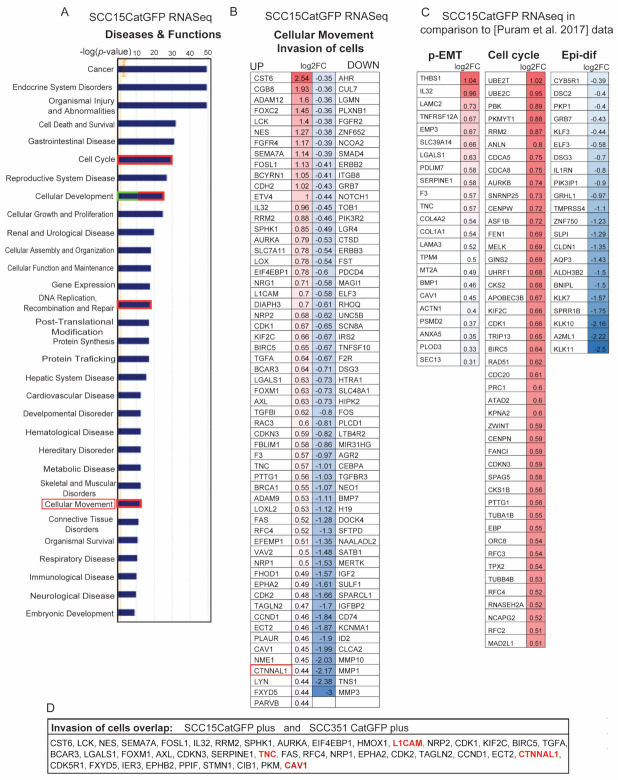
Signature genes of invasive Catulin-GFP reporter-labeled cancer cells. (**A**) Bar chart presenting the most changed processes in the sorted SCC15CatGFP plus population. (**B**) Heatmap of genes changed in the sorted SCC15CatGFP plus population associated with the invasion of cells. (**C**) Heatmaps of genes changed in the sorted SCC15CatGFP plus population associated with the cell cycle, partial epithelial–mesenchymal transition (p-EMT), and epithelial differentiation (Epi-dif) in comparison to genes presented by [Puram et al., 2017] [30]. Catenin alpha-like 1 (CNTTAL1) gene is boxed. (**D**) Table presenting upregulated genes common for the SCC15CatGFP plus and SCC351CatGFP plus populations involved in the in invasion of cells. CTNNAL1 and other genes of interest are highlighted in red.

To determine the genetic signature of Catulin-GFP reporter-positive cells, we performed RNAseq analysis and compared sorted SCC15CatGFP+/alpha6+ GFP-positive cells (invasive cells) and SCC15CatGFP−/alpha6+ (non-invasive) cancer cells (Figure 3B,C). The principal component analysis (PCA) of RNAseq analyses for the sorted SCC15CatGFP plus (GFPp) and SCC15CatGFP minus (GFPn) populations are presented in Supplementary Figure S1E. The proper function of the reporter system and the sorting strategy were verified by the appearance in the RNAseq data from tumors formed after injection of both cell lines, *CTNNAL1* gene in SCC15CatGFP plus populations as an internal control (boxed in Figure 4B and highlighted in red in Figure 4D). The gene ontology enrichment analysis of biological processes revealed an increase in general categories such as cancer, endocrine system disorders, organismal injury and abnormalities, as well as the cell cycle, cellular development, DNA replication, and cellular movement (Figure 4A and Supplementary Figure S3A,B). We focused our further analysis on genes involved in cellular movement and subcategories and the invasion of cells (boxed in Figure 4A and boxed in Supplementary Figure S3A). This category included 59 upregulated genes of particular interest because of their potential role in tumor invasion (*p* < 0.05; Figure 4B). We next wanted to verify if data obtained in our screen correlated with clinical data. Therefore, we used data published by Puram et al. [30], in which they profiled transcriptomes of ~6000 single cells from 18 HNSCC patients, including five matched pairs of primary tumors and lymph node metastasis. Although malignant cells from human specimens varied within and between tumors, they established signatures of common genes for the partial epithelial-to-mesenchymal transition (p-EMT), cell cycle, and epithelial differentiation (Epi-dif) among others. The comparison of our RNAseq data with the established human specimen signatures revealed a strong overlap (23%) of upregulated genes important for p-EMT and (45%) for the cell cycle. As expected for cells going through a p-EMT, the genes important for epithelial differentiation were mainly downregulated in our screen, which correlated with the data obtained from human specimens (22% overlap; Figure 4C). The comparison of the data obtained using two independent reporter cell lines, namely SCC15CatGFP and SCC351CatGFP within the most interesting category of cell invasion, revealed that many genes belonging to this category were changed in both cell lines (Figure 4D). It is important to emphasize genes upregulated in both cell lines, namely *CATULIN*, L1 cell adhesion molecule (*L1CAM*), *FOXM1*, *SERPINE1*, neuropilin-1 (*NRP1*), *EPHA2*, tenascin-C (*TNC*), and caveolin-1 (*CAV1)*, suggesting a common mechanism of invasion in HNSCC.

### 2.5. Genetic Signature of Catulin-GFP Reporter-Labeled Cancer Cells Indicates an Enrichment in Genes Involved in Axonal Guidance, Glioblastoma Multiforme, ILK, and Integrin Signaling Pathways

**Figure 5:**
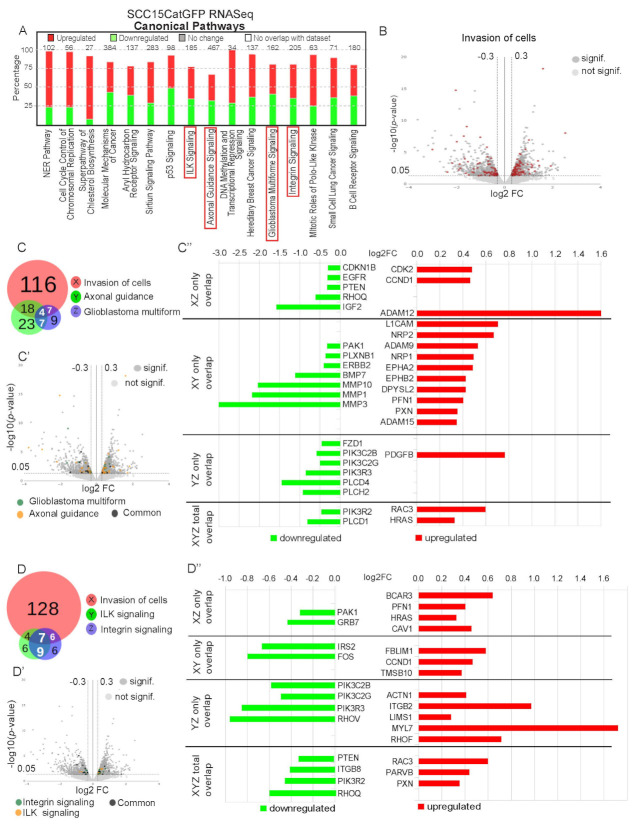
Ingenuity pathway analysis results of RNAseq data of invasive Catulin-GFP reporter-labeled cancer cells. (**A**) Box chart presenting the most changed signaling pathways in the sorted SCC15CatGFP plus population. Signaling pathways of interest are boxed (integrin-linked kinase (ILK), axonal guidance, glioblastoma multiforme, and integrin signaling). (**B**) Volcano plot showing the fold change (log2FC) and statistical significance (*p*-value) for all genes differentially expressed in the sorted SCC15CatGFP population. Genes shown in dark grey were statistically significant (*p* < 0.05 and log2FC > 0.3), genes shown in light grey were statistically insignificant (*p* > 0.05 and log2FC < 0.3), and genes shown in red were significant genes involved in the invasion of cells. (**C**) BioVenn diagram showing the overlap between genes involved in the invasion of cells (X, red), genes related to axonal guidance signaling (Y, green), and genes related to glioblastoma multiforme signaling (Z, violet). The numbers inside the diagram indicated the number of genes in each group. The area of the overlap was proportional to the number of common genes. (**C′**) Volcano plot showing the fold changes (log2FC) and statistical significance values (*p*-value) for all genes differentially expressed in the sorted SCC15CatGFP population. Genes shown in dark grey were statistically significant (*p* < 0.05 and log2FC > 0.3), genes shown in light grey were statistically insignificant (*p* > 0.05 and log2FC < 0.3), genes shown in green were significant genes involved in axonal guidance, genes shown in orange were significant genes involved in glioblastoma multiforme signaling, and genes shown in black were significant genes common for axonal guidance and glioblastoma multiforme signaling. (**C″**) Bar chart presenting genes significantly (*p* < 0.05 and log2FC > 0.3) downregulated (in green) and upregulated (in red) that belonged to more than one group (common for X/Z, X/Y, Y/Z, or X/Y/Z). (**D**) BioVenn diagram showing the overlap between genes involved in the invasion of cells (X, red), genes related to ILK signaling (Y, green), and genes related to integrin signaling (Z, violet). The numbers inside the diagram indicated the number of genes in each group. The area of the overlap was proportional to the number of common genes. (**D′**) Volcano plot showing the fold changes (log2FC) and the statistical significance values (*p*-value) for all genes differentially expressed in the sorted SCC15CatGFP population. Genes shown in dark grey were statistically significant (*p* < 0.05 and log2FC > 0.3), genes shown in light grey were statistically insignificant (*p* > 0.05 and log2FC < 0.3), genes shown in green were significant genes involved in integrin signaling, and genes shown in orange were significant genes involved in ILK signaling xD (log2FC > 0.3) and downregulated (in green) and upregulated (in red) that belonged to more than one group (common for X/Z, X/Y, Y/Z, or X/Y/Z). (**D″**). Bar chart presenting genes significantly (*p* < 0.05 and log2FC > 0.3) downregulated (in green) and upregulated (in red) that belong to more than one group (common for: X/Z, X/Y, Y/Z or X/Y/Z).

To obtain a deeper insight into pathways that can be involved in the increased invasive potential of the SCC15CatGFP plus population, we performed ingenuity pathway analysis. Among the signaling pathways most changed in the SCC15CatGFP plus population, axonal guidance, glioblastoma multiforme, ILK, and integrin signaling pathways received special attention in the light of cancer–stroma interactions during invasion and metastasis (Figure 5A). We analyzed 145 changed (up- or downregulated) genes in the SCC15CatGFP plus population that was associated with the invasion of cells and compared them with the list of changed genes involved in axonal guidance and glioblastoma multiforme signaling pathways. Out of 145 genes involved in invasion, 18 overlapped with axonal guidance signaling, seven genes overlapped with glioblastoma multiforme signaling, and four genes were common for all three gene categories (Figure 5B,C,C′,C″).

Interestingly, L1CAM, of which the upregulation correlated with the unfavorable prognosis for HNSCC patients is also upregulated in Catulin-GFP plus populations. L1CAM is a neural adhesion transmembrane glycoprotein belonging to the immunoglobulin superfamily that plays a crucial role in nervous system development, and its expression level is elevated in many cancers, promoting cancer cell motility and invasion and in some types of cancer is associated with PNI. Additional genes involved in axonal guidance, NRP1 and neuropilin-2 (NRP2), are also elevated in SCC15CatGFP plus populations. It is important to emphasize that the direct interaction of L1CAM with NRP1 plays a crucial role in this process. Neuropilins also play important role in tumor vascularization, promoting metastasis, and their co-expression correlates with increased vascularity and poor prognosis for NSCLC patients.

Another group of genes that are upregulated in SCC15CatGFP plus populations are ephrin receptors, i.e., EphA2 and EphB2. Studies concerning their functions in cancer suggest their roles in cell growth, survival, migration tumor initiation, and angiogenesis, and their overexpression levels are correlated with aggressiveness, poor prognosis, and metastasis in various cancers.

We performed an analogical comparison of 145 genes involved in invasion with ILK and integrin signaling pathways affected genes. Four genes involved in invasion overlapped with ILK signaling, six genes overlapped with integrin signaling, and seven genes were common for all three gene categories (Figure 5D,D′,D″). The most upregulated genes that were associated with the invasion of cells and ILK and integrin signaling are breast cancer antiestrogen resistance 3 (BCAR3), Cav1, α-actinin-1 (ACTN1), paxillin (PXN), and parvin-β (PARVB), with most of them being involved in cytoskeleton organization, cell adhesion, and focal contacts formation. Interestingly, specific Rho molecules showed to be affected, with RhoF being upregulated whereas RhoV and RhoQ being downregulated in the SCC15CatGFP plus cells. Our RNAseq analysis also revealed that in the SCC15CatGFP plus population, there was an upregulation of two semaphorins, SEMA7A and SEMA6B (Supplementary Figure S4A), which are proteins that take part in the nervous system development by axonal guidance. One of the SEMA7A receptors is PlexinC1, a transmembrane protein that can act as a tumor suppressor through the inhibition of cofilin 1 (CFL1), an actin-binding protein that play role in cell migration. Our RNAseq data showed that in the SCC15CatGFP plus population PlexinC1 expression was downregulated (log2FC: −3.749; *p*-value: 1,76 × 10^−6^), and the expression of CFL1 was upregulated (log2FC: 0.3; *p*-value: 0.019), what indicated an increased migratory potential of the SCC15CatGFP plus population.

The comparison of the data obtained using two independent reporter cell lines SCC15CatGFP and SCC351CatGFP within most interesting categories, i.e., cell invasion, axonal guidance, glioblastoma multiforme, ILK, and integrin signaling pathways, revealed that many genes belonging to those categories were changed in both cell lines (Supplementary Figure S5A).

To confirm the clinical importance of our findings, we correlated our RNAseq data with the data collected in the Human Protein Atlas for genes associated with poor prognosis for the HNSCC patients. We found that 12 genes that are listed as unfavorable for patients overlapped with one or more categories changed in the SCC15CatGFP plus cells (10 with invasion of cells, three with axonal guidance, two with glioblastoma multiforme signaling, three with ILK signaling, and three with integrin signaling; Supplementary Figure S5B). Moreover, nine of these genes are common for SCC15CatGFP and SCC351CatGFP. The other important process that is involved in cancer metastasis is a degradation of the ECM. The analysis of our RNAseq data showed the upregulation of five of a disintegrin and metalloproteinases ADAMs (ADAM12, ADAM19, ADAMS16, ADAM9, and ADAM15) in the SCC15CatGFP plus population (Supplementary Figure S4B). This might suggest that SCC15CatGFP plus cells have a higher ability to degrade the ECM and as a result, more effectively invade the surrounding stroma in comparison to the SCC15CatGFP minus population.

### 2.6. Known Invasion Markers PXN and Tenascin-C Are Expressed in the Cells Marked by the Catulin Reporter System at the Tumor Invasion Front

**Figure 6:**
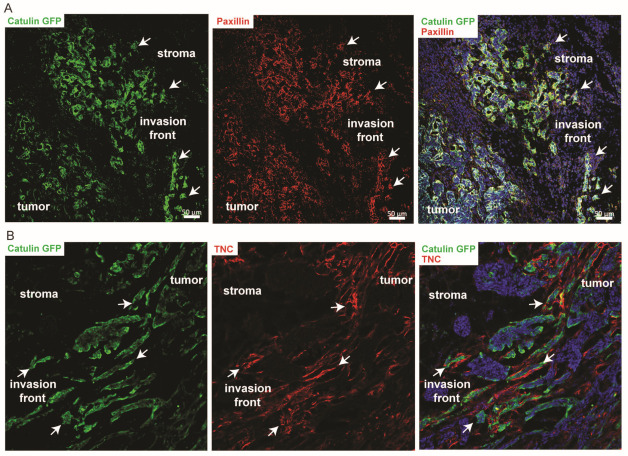
Cell invasion markers localization in Catulin-GFP reporter tumors. (**A**) Immunofluorescence staining of the section of tumor formed after the injection of SCC15CatGFP and the use of the paxillin antibody. GFP (catulin reporter) immunofluorescence is indicated in green, paxillin immunofluorescence is indicated in red, and DAPI (nucleus) immunofluorescence is indicated in blue. Arrows indicate cells expressing high catulin reporter and paxillin levels. Scale bar: 50 µm. (**B**) Immunofluorescence staining of the section of tumor formed after the injection of SCC15CatGFP and the use of the tenascin C (TNC) antibody. GFP (catulin reporter) immunofluorescence is indicated in green, TNC immunofluorescence is indicated in red, and DAPI (nucleus) immunofluorescence is indicated in blue. Arrows indicate cells expressing high catulin reporter levels and tenascin C levels.

We first performed immunofluorescence stainings with known markers of invasion, namely TNC and PXN, which appeared in our RNAseq screen, as genes involved in the invasion. TNC is an adhesion modulatory ECM molecule that is highly expressed in the microenvironment of most solid tumors. It is a factor in the tumor-specific microenvironment that is expressed by both transformed epithelial cells and stromal cells. TNC is a key determinant of the tumor stroma that is involved in the initiation of tumorigenesis and progression to metastasis. We observed a strong upregulation of TNC correlating with GFP fluorescence signals from the catulin reporter expressing tumor cells (arrows in Figure 6B). PXN is a focal adhesion adapter protein with an important scaffolding role at focal adhesions by recruiting structural and signaling molecules involved in cell movement and migration. As a major participant in the regulation of cell movement through integrins, PXN plays distinct roles in normal development and homeostasis. Importantly, PXN is also an essential player in pathological conditions including cancer development and metastasis. In tumors derived from the injection of catulin reporter cell lines, a strong expression level of PXN was visible at the tumor invasion front, co-localizing with catulin-GFP expression (arrows in Figure 6A).

### 2.7. CAV1 Is Expressed at the Invasion Front in Tumors Derived from Cells with the Catulin Reporter System and in the Human Specimens of HNSCC

**Figure 7:**
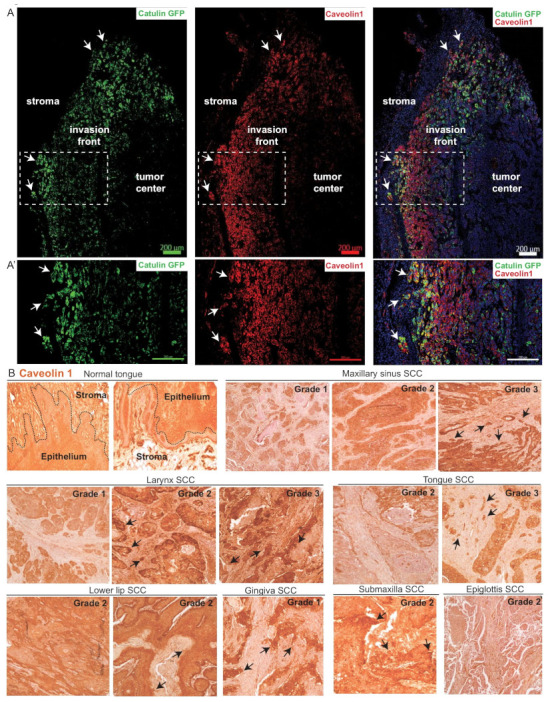
Caveolin-1 localization in Catulin-GFP reporter tumor and in human HNSCC cancer tissues. (**A**) Immunofluorescence staining of the section of tumor formed after the injection of SCC15CatGFP and with the use of the caveolin-1 antibody. GFP (catulin reporter) immunofluorescence is indicated in green, caveolin-1 immunofluorescence is indicated in red, and DAPI (nucleus) immunofluorescence is indicated in blue. The boxed area in (**A**) are enlarged in (**A′**). Arrows indicate the co-localization of the catulin reporter with caveolin-1. Scale bar: 200 µm. (**B**) Immunohistochemical DAB stainings of caveolin-1 in a tissue array of HNSCC samples and in normal human tongue tissue. Arrows indicate high caveolin-1 expression in potentially invasive cells. Tissue origin and tumor grade, as provided by the manufacturer, are indicated. The stroma/epithelium border in normal tongue tissue is indicated.

In cancer, CAV1 operates as both a tumor suppressor gene and a promoter of metastasis, depending on the cancer type and stage [31,32]. Therefore, we wanted to assess the expression of CAV1, because it was upregulated in tumors formed after injection of both reporter cell lines. We confirmed the enrichment and co-localization of CAV1 protein, with the GFP expression of labeled cells at the invasion front of the SCC15CatGFP tumors (Figure 7A,A′). Cells with a high catulin reporter GFP expression at the tumor invasion front were also positive for CAV1 protein (arrows in Figure 7A,A′). The high expression levels of both catulin and CAV1 proteins were visible in streams/clusters of cancer cells invading the stroma. (arrows in Figure 7A′). To verify if the expression of CAV1 protein correlated with the tumor stage of different HNSCC cells, we performed immunohistochemical analysis of CAV1 on a human tissue microarray slide panel (Figure 7B). We compared the expression levels of CAV1 in different stages of maxillary sinus SCC, larynx SCC, and tongue SCC. Higher CAV1 expression was observed in grade 2 and/or grade 3 of maxillary sinus, larynx, tongue, and lower lip SCC as compared to in grade 1. Interestingly, a gingiva SCC expression level of CAV1 was observed even in grade 1 (Figure 7B). For comparison, the normal tongue epithelium showed some level of CAV1 expression, but at a much lower level than in cancer tissues. We concluded that the expression of CAV1 correlated with the progression of the tumor. In advanced stages of cancer, we observed areas with strong CAV1 staining, indicating its high expression, especially in cancer cells invading neighboring stroma (arrow in Figure 7B).

### 2.8. Invasive Cells Marked by the Catulin Reporter System and Human Specimens of HNSCC Express a Perineural Invasion Marker L1CAM at the Tumor–Stroma Border

**Figure 8:**
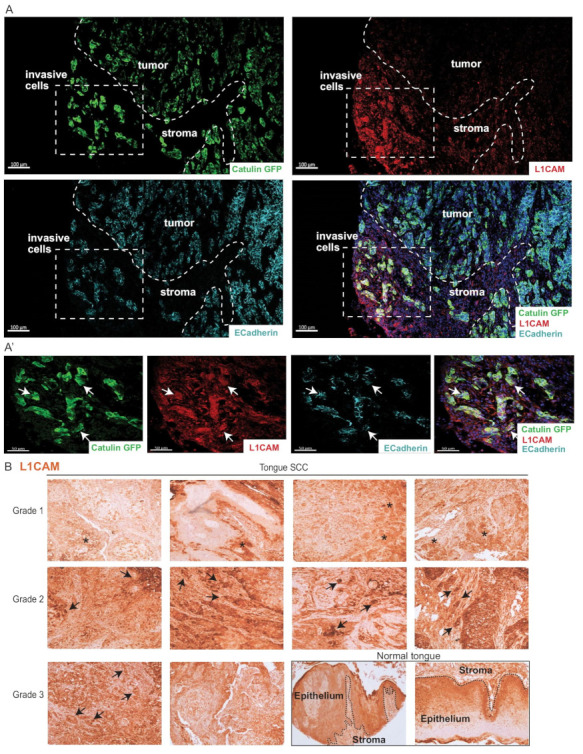
L1 cell adhesion molecule (L1CAM) localization in Catulin-GFP reporter tumor and in human HNSCC cancer tissues. (**A**) Immunofluorescence staining of the section of SCC15CatGFP tumor formed with the injection of SCC15CatGFP and with the use of the L1CAM antibody. GFP (catulin reporter) immunofluorescence is indicated in green, L1CAM immunofluorescence is indicated in red, E-cadherin immunofluorescence is indicated in cyan, and DAPI (nucleus) immunofluorescence is indicated in blue. The boxed area in (**A**) are enlarged in (**A′**). Arrows indicate the co-localization of the catulin reporter with L1CAM. The dashed line indicates the tumor–stroma border. Invasive cells are indicated. Scale bar: 200 µm. (**B**) Immunohistochemical DAB stainings of L1CAM in a tissue array of tongue SCC samples and in normal human tongue tissue. Arrows indicate high L1CAM expression in potentially invasive cells. Asterisks indicate regions with A higher than average level of L1CAM expression. Tumor grade, as provided by the manufacturer, is indicated. The stroma/epithelium border in normal tongue tissue is indicated.

The RNAseq data obtained using our reporter system indicate the upregulation of genes involved in axonal guidance signaling. L1CAM is a neuronal adhesion molecule that also plays a role in perineural invasion. L1CAM is shown to be involved in proliferation, invasion, and metastasis of different tumor types and is engaged in homophilic interactions with many other ligands in a context-dependent manner. To verify the enrichment and co-localization of L1CAM with the expression of GFP-labeled cells at the invasion front of the SCC15CatGFP tumors, immunofluorescence staining with the anti-L1CAM antibody was performed (Figure 8A,A′). We observed that GFP-positive clusters of invasive cells at the tumor invasion front expressed high levels of L1CAM (Figure 8A,A′). High expression levels of catulin-GFP and L1CAM correlated with low levels of E-cadherin (arrows in Figure 8A′), indicating cells going through a partial EMT. To asses if the expression of L1CAM correlated with the clinical tumor stage of tongue SCC, we performed immunohistochemical stainings of L1CAM on a tissue microarray slide panel (Figure 8B). In the majority of advanced stages of human tongue cancers, clusters of cells strongly positive for L1CAM were visible (arrows in Figure 8B). On the contrary, the expression level of L1CAM in grade 1 cancers was much lower, except for some parts of cancer tissue where the expression level of L1CAM was slightly higher (asterisks in Figure 8B); however, this level of expression was comparable to in normal tongue tissue.

## 3. Discussion

HNSCC is a common malignancy with high and unchanged death rates for decades. In comparison to standard surgery with resection margins of >1 cm, compartmental tongue surgery (CTS) improves disease-free and overall survival, indicating the importance of tumor–stroma interactions in the locoregional spread of this type of cancer [33]. In HNSCC, an EMT cell state is associated with cancer aggressiveness and poor prognosis. However, the transient and reversible nature of the EMT process involved in tumor invasion precludes the use of surface markers to effectively track, isolate and characterize invasive cancer cells in vivo, enhancing our understanding of their biology. Our previous investigation concerning the pathogenesis of HNSCC showed that cells at the tumor invasion front had a high expression of catulin protein and that this protein might be involved in cancer metastasis. Therefore, to label, track and characterize cells with higher catulin expression and invasive potential, we developed a novel reporter system based on GFP protein expression under the short catulin promoter. Based on GFP fluorescence, which indicated catulin expression, we sorted and analyzed cells from the Catulin-GFP reporter cell line, showing their increased migratory, invasive, and tumorigenic potential. These results are in accordance with our previous findings from knock-out experiments, where we showed that the ablation of catulin expression resulted in a decrease of migratory and invasive potential of hHNSCC cells in vitro and in vivo [25].

As expected, the analysis of tumors formed after the injection of Catulin-GFP reporter cells revealed an opposite correlation in the expression between varying levels of an epithelial marker, E-cadherin and Catulin-GFP reporter, and in cells localized predominantly at the tumor invasion front, indicating that those cells underwent a partial EMT. The partial decrease or destabilization of E-cadherin at the cell–cell contacts only at the tumor invading front indicated metastasis through collective cell migration and invasion, which is very common for HNSCC. This phenomenon occurs, when a group of cells with preserved cell–cell contacts invade and migrate cohesively as a multicellular unit conducted by leader cells, which consists of stromal CAFs and invasive cancer cells [34]. Although it is well-established that collectively migrating cells exhibit homotypic cell–cell interactions, but a less-appreciated is the contribution of heterotypic cell–cell interactions between cancer and stromal cells [35]. More recently, the heterotypic adhesion between CAFs and cancer cells using N-cadherin and E-cadherin is shown to lead to CAF-led collective cell migration. Stromal CAFs can also facilitate invasion by remodeling of the ECM to clear an area for invading cancer cells as was observed in SCC [35]. It could be an explanation for the lack of the upregulation of typical ECM degrading enzymes, Matrix metalloproteinases (MMPs) in the invasive SCC15CatGFP plus population. However, there was an upregulation of five disintegrin and metalloproteinases ADAMs: ADAM12 that can degrade gelatin, fibronectin, and type IV collagen [36], ADAM9 that degrades fibronectin and collagen XVII, ADAM15 degrading type IV collagen and gelatin [37], ADAMTS16 that can degrade aggrecan [38], and ADAM19 that is described as a marker of the EMT [39] (Supplementary Figure S4B). The p-EMT and collectively migrating cell clusters with a mixed phenotype of epithelial (e.g., adhesion) and mesenchymal (e.g., migration) properties have many advantages over single migrating cells, which in general results in much higher tumor-initiating and metastatic potential, as well as in resistance to chemotherapy and immune systems and overall apoptosis [40]. This model can be supported by our RNAseq analysis of SCC15CatGFP plus cancer cells, which revealed an increase in the invasion of cells, cell proliferation, cell cycle progression, and cell survival and viability, and a decrease in cell death, necrosis, and apoptosis.

The RNAseq data also indicated that the SCC15CatGFP plus population was enriched with genes involved in the progression of the cell cycle. We hypothesized that it could be due to the misregulation of three genes engaged in cell cycle regulation—cyclin dependent kinase 2 (CDK2) and G1/S-specific cyclin D1 (CCND1), which are upregulated and cyclin-dependent kinase inhibitor 1B (CDKN1B) that is downregulated. An elevated level of CCND1 is correlated with poor prognosis, and its expression is upregulated in 30% to 50% of HNSCC patients due to gene amplification [41,42]. CDK2, which is also an unfavorable prognosis gene, can be associated with radiotherapy resistance [43]. The activities of both CDK2 and CyclinD1 can be inhibited by CDKN1B, which is downregulated in the SCC15CatGFP plus population. The exact mechanism of CDKN1B downregulation in our study is not known, but it could be possible through miR-196a delivered by exosomes released by CAFs [44]. This scenario could be possible because of the proximity of the SCC15CatGFP plus population to the TME and CAFs, as those cells localize mainly at the tumor invasion front. Overall, these findings suggest that both the expression level and activity of CDK2 and CyclinD1 could be elevated, which can lead to increased cell cycle progression and cell proliferation.

The noticed upregulation of genes involved in the ILK and integrin signaling pathways in Catulin-GFP reporter cells emphasized previously published potential mechanism of action for catulin in cancer metastasis by activating the ILK-mediated Akt-NF-κB-αvβ3 signaling axis [29]. The analysis of our RNAseq data also showed an upregulation of the BCAR3 gene, engaged in enhanced cancer cell motility, by regulation of the balance between Rac1 and RhoA signaling in invasive breast cancer cells [45]. The Catulin-GFP plus population also showed a upregulation of ACTN1 gene, of which the high expression level was recently associated with the clinical stage, node metastasis, and poor prognosis for oral squamous cell carcinoma (OSCC) patients [46]. PARVB, a direct binding partner of ACTN1 [47] and ILK, is also upregulated in the SCC15CatGFP plus population. PARVB is involved in mediating actin polymerization in focal adhesions. Therefore, it plays an important role in promoting cell spreading [48] and was shown to be overexpressed in tongue SCC [49]. PARVB can also bind directly and specifically to PXN, another focal adhesion protein upregulated in the SCC15CatGFP plus population. This interaction between PARVB and PXN is crucial for the early recruitment and proper localization of PARVB to focal adhesions [50]. PXN is also one of the unfavorable prognosis genes in HNSCC [51,52].

High and unchanged mortality of HNSCC patients for decades is mainly caused by tumor dissemination and metastasis. This process can occur through the blood and lymphatic system as well as along the nerves by PNI mediated by factors that normally play role in nervous system development such as axon guidance molecules. Our RNAseq analysis of the invasive SCC15CatGFP plus population revealed the upregulation of genes involved in axonal guidance (Figure 5C′). One of the genes involved in axonal guidance is L1CAM, of which the expression correlates with aggressive clinical features [53] as well as poor prognosis in many types of cancer [54]. It was also reported that L1CAM expression is correlated with perineural invasion and poor outcomes in pancreatic ductal adenocarcinoma [55,56]. L1CAM protein can interact with NRP1 by direct association through their extracellular domains what is required for axon guidance [57]. The expression level of NRP1 is elevated in the SCC15CatGFP plus population as well as the expression of second subtype, NRP2. Neuropilins were also shown to engage in tumor neovascularization and metastasis, and their co-expression was shown to be correlated with the increased vascularity and poor prognosis for NSCLC patients [58]. The expression of both NRP1 and NRP2 was found in many tumor types [59], where their overexpression contributed to metastasis [59,60,61].

In our screen, we also showed a upregulation of semaphorin7A (Sema7A). This membrane-linked semaphorin promotes the outgrowth of central and peripheral axons. Sema7A has pro-angiogenic properties [62], can contribute to the metastasis of breast cancer cells by inducing EMT [63] and was reported to be overexpressed in oral SCC, where the proliferation and invasiveness of cancer cells are enhanced [64]. Sema7A can bind to its receptor Plexin C1 (PLXNC1). In melanoma, it was shown that PlexinC1 is a tumor suppressor protein, as it inactivates CFL1, an actin-binding protein critical for cell adhesion and migration. In metastatic melanoma, there is a significant loss of PLXNC1, which results in an enhanced activation of CFL1 and acquisition of a metastatic phenotype by cancer cells [65,66]. Our data indicated a possibility of a similar model as, apart from the upregulation of Sema7A, there were a downregulation of PLXNC1and a upregulation of CFL1 in the SCC15CatGFP population.

The function of some genes upregulated in the Catulin-GFP reporter cells in cancer progression is still controversial such as for Cav1, which can act as both tumor suppressor, by regulating integrin β1- and Src-mediated cell–cell and cell–matrix interactions [67,68,69] and tumor-promoting molecules by affecting miR-133a [70,71]. However, our data from human HNSCC tissue arrays suggested that the expression of CAV1 correlated with the progression of tumor and was specifically stronger in advanced stages of cancer, especially in cancer cells invading neighboring stroma.

Importantly, the comparison of our RNAseq data with data published by Puram et al. [30], in which they profiled transcriptomes of ~6000 single cells from 18 HNSCC patients, including five matched pairs of primary tumors and lymph node metastasis, revealed a correlation within the established signatures of common genes for the partial EMT, cell cycle, and epithelial differentiation. This comparison strengthened our findings, which provided an even longer list of potential genes involved in HNSCC invasion. Our reporter system had a probably higher sensitivity because of the labeling and sorting out a very small, specific population of invasive cells from the tumor. In addition, the comparison of the results from tumors derived from two different HNSCC catulin reporter cell lines showed a pool of genes associated with invasion and common for both cell lines, suggesting similarities in invasiveness and metastasis. Those genes, among others, were *Sema7A*, *L1CAM*, *NRP1*, *NRP2*, *BCAR3*, *IER3*, *CAV1*, *EPHA2*, *EPHB2*, *CCND1*, *CDK2*, *and CTNNAL1* (catulin).

## 4. Materials and Methods

### 4.1. Cell Lines and Generation of the Catulin Reporter System

An SCC15 cell line was obtained from the American Type Culture Collection (ATCC, Manassas, VA, USA) and authenticated by ATCC with tests such as short tandem repeat profiling (STR profiling). SCC351 (USC-HN1) was previously described and characterized [72]. Both cell lines were cultured in Dulbecco’s modified Eagles’s medium (DMEM; high glucose; Biowest, Nuaillé, France, #L0102-500) containing 10% fetal bovine serum (FBS) (Biowest, #S181S-500), 1% antibiotics: penicillin (100 U/mL), and streptomycin (100 µg/mL) (Biowest, #L0018-100). The cell line was grown at 37 °C in humidified 5% CO_2_/95% air atmosphere. The number of living cells was calculated by trypan blue staining using a EVETM Automatic Cell Counter (Nano EnTek, Seoul, Korea). The cell line was regularly tested for mycoplasma contamination using a PCR-based method [73]. To generate stable reporter cell lines, SCC15 and SCC351 cell lines were transfected with GLuc-ON Promoter Reporter Clone (#HPRM14050-PF02; GeneCopoeia, Rockville, MD, USA) construct using Lipofectamine 3000 Transfection Reagent (#L3000001; Thermo Fisher Scientific, Waltham, MA, USA) according to the manufacturer’s protocol. 48 h after transfection, the cells were treated with puromycin to select properly transfected cells and establish stable cell lines. For GFP fluorescence analysis, an ArthurTM (Nano EnTek, Seoul, Korea) Fluorescence Cell Counter was used. Detailed catulin plasmid informations are provided in Supplementary Table S1. 

### 4.2. FACS and Flow Cytometry Fluorescence Analysis

Flow cytometry fluorescence analysis and FACS was performed using BD FACS, Becton, Dickinson and Company, San Jose, CA, USA Aria Fusion. SCC15CatGFP cells were trypsynized, fixed and permabilized, and single cells suspensions in 1% FBS (free Ca^2+^) in dPBS were stained with Alpha6 Integrin (conjugated to PE) in a 1:200 dilution for 30 min in the dark on ice. Cell viability was determined by adding DAPI in a 1:1000 dilution before cell sorting. The samples were analyzed using Diva software (Version 8.0.1, BD Biosciences, San Jose, CA, USA). Gates for cell sorting were set in relation to the unstained control SCC15 cell line. A similar protocol was used for the SCC351 cell line.

### 4.3. Protein Isolation and Western Blot Analysis

The cells were washed with phosphate-buffered saline (PBS) and scraped in an NP-40 lysis buffer supplemented with 1% protease inhibitor and 1% phosphatase inhibitor cocktails. Proteins were separated by electrophoresis in 10% SDS-PAGE gels and then electro-transferred onto nitrocellulose membranes. The membranes were blocked by incubation with 5% non-fat milk in TBS (0.1% Tween-20) for 1 h at room temperature and then incubated overnight at 4 °C with primary antibodies. Next day, the membranes were incubated with appropriate HRP-conjugated secondary antibodies. Signals were visualized by enhanced chemiluminescence detection (SuperSignal^®^West Dura, Thermo Scientific™, Waltham, MA, USA). As a loading control, the membranes were incubated with a monoclonal anti-GAPDH antibody.

### 4.4. Sphere Invasion

In order to form spheres from the SCC15CatGFP cell line, 100 cells per well in a 96-well low-attachment plate were grown in sphere formation media for one week. After that, the spheres were gently transferred into an 8-well chamber slide (IBIDI) filled with Matrigel (BD Biosciences, San Jose, CA, USA) or rat-tail collagen and cultured for additional three days. Images of invading spheres were taken under a fluorescence microscope.

### 4.5. Migration and Invasion Assay

Cell migration and invasion were determined using a Boyden insert chamber (8-µm pore size) and a BD BioCoat (Bedford, MA, USA) Matrigel Invasion Chamber (8 µm), respectively. Cells were trypsynized and resuspended in a serum-free medium and then counted with an EVE^TM^ Automatic Cell Counter (Nano EnTek). A total of 100,000 cells were resuspended in a DMEM medium, were seeded inside the insert and were incubated for 24 h at 37 °C in a humidified 5% CO_2_ atmosphere. Outside the insert, a DMEM medium supplemented with 10% FBS was used as the chemoattractant. The cells that migrated or invaded through the membrane of chamber were fixed and stained using crystal violet. Experiments were repeated at least three times.

### 4.6. Colony Formation Assay

To investigate the colony formation ability, an equal number of cells (150, 300, and 600 cells per well) was plated in triplicates into six-well plates. The colonies were visualized after 10 days of culturing using a crystal violet dye. Experiments were repeated at least three times.

### 4.7. Xenograft Transplants and Experimental Animals

A total of 1 × 10^6^ cells suspended in media were mixed with Matrigel (BD Biosciences) at a volume ratio of 1:1 and injected subcutaneously between the neck and shoulder of NOD SCID mice. Tumors were allowed to form for 4 to 9 weeks before sacrificing and collecting the primary tumor. For further analysis and FACS, three independently formed tumors were taken. Mice were maintained and bred in Central Laboratory of Experimental Animals, Medical University in Warsaw in the Individually Ventilated Cages (IVC) system.

### 4.8. Dissection Scope Pictures

Images of isolated tumors formed after the injection of SCC15CatGFP cell line were taken under a Leica (Wetzlar, Germany) dissection scope.

### 4.9. Tissue Preparation and Cell Isolation

All steps of cell isolation from tissue were performed on ice. Mice tongues were placed on petri dishes and chopped into small pieces using scissors and a razor blade in a small volume of a cold culture medium. The soup was transferred to a 50 mL tube filled with 20 mL cold culture medium and spun at 300× *g* for 5 min at 4 °C. The supernatant was discarded, 20 mL of cold dPBS were added, and the samples were spun at 300× *g* for 5 min at 4 °C. The supernatant was discarded, and the pellet was resuspended in 1 mL of collagenase I solution (1000 U of collagenase/mL media). The samples were incubated at 37 °C for 45 min with gentle agitation. After incubation, the samples were spun at 300× *g* for 5 min at 4 °C, the supernatant was discarded, and the pellet was resuspended in a 0.25% trypsin solution. The samples were incubated at 37 °C for 15 min with gentle agitation. After neutralizing trypsin activity by adding 15 mL of cold media with FBS and spinning the samples at 300× *g* for 5 min at 4 °C, the pellet was resuspended in 15 mL of low calcium media and cell suspension was passed on pre-wetted 70 μm and 40 μm nylon meshes. The samples were spun at 300× *g* and at 4 °C 5 min and resuspended in 1% FBS (free Ca^2+^) in dPBS. Single cells suspensions were used for FACS.

### 4.10. Hematoxlin and Eosin (H&E) Staining and Immunofluorescence

Tissues for immunofluorescence and H&E staining were incubated in sucrose solution for 1 hour in room temperature, fixed overnight in 4% paraformaldehyde (PFA), embedded in an OCT compound and frozen at −80 °C. Frozen tissues were cut on Leica cryostat, and 12 μm-thick cryosections were kept at −80 °C until required for staining. H&E staining was performed according to a standard protocol. Tissues for immunofluorescence staining were fixed in 4% PFA for 10 min at room temperature and subsequently permeabilized in 0.1% Triton X-100 in PBS (PBS-T) for 10 min at room temperature. Non-specific sites were blocked in 0.1% bovine serum albumine (BSA), 2.5% normal goat serum (NGS), and 2.5% normal donkey serum (NDS) in 0.1% PBS-T for 30 min at room temperature. Specific primary antibodies were diluted in 0.1% BSA in PBS-T and incubated overnight at 4 °C. Next day, secondary fluorochrome-conjugated antibodies were diluted at 1:500 in a blocking solution and incubated 1 h at room temperature in the dark. For actin visualization, the samples were incubated with phalloidin-TRITC at a dilution of 1:200 for 1 h in room temperature in the dark. The samples were counterstained with nuclear dye DAPI at a dilution of 1:1000 for two minutes at room temperature in the dark. The samples were mounted in FluorSave (Merck Millipore, Burlington, MA, USA). Images were acquired by a laser scanning confocal microscope LSM 700 (Zeiss, Oberkochen, Germany) and analyzed with Zen2 Program (Zeiss, Zen 2012 SP5 FP3 (black ) Version 14.0.17.201; Zen 2.3 (blue) Version 2.3.64.0). The information about antibodies used for immunofluorescence is presented in Supplementary Table S1.

### 4.11. Immunohistochemistry

The immunohistochemical staining analysis for CAV1 and L1CAM was performed on tissue microarray slide panels HN483 and OR601a (Biomax, Derwood, MD, USA), respectively. The slides were deparaffinized and rehydrated with a standard protocol, and heat-induced antigen unmasking was performed using a special antigen-unmasking retriever in 0.01 M sodium citrate (pH 6) for 10 min at 120 °C. After blocking the endogenous peroxidase activity in 0.3% H_2_O_2_ for 3 min, the slides were blocked in a blocking buffer (0.1% gelatin, 0.1% BSA, 2.5% donkey serum, 2.5% goat serum, and 0.3% Triton X in PBS) for 1 h at room temperature. After that, the slides were incubated with a primary antibody in 0.1% BSA in 0.1% PBS-T ON at 4 °C. Next day, the slides were incubated in appropriate biotin-conjugated secondary antibody (1:100) (Vector Labs, Burlingame, CA, USA) for 1 h in the blocking buffer. Next, the slides were incubated in the prepared Vectastain A + B solution (2 drops of A, 2 drops of B, 2.5 mL PBS 1X) for 30 min at RT. Reactions were developed using the diaminobenzidine (DAB) reagent as the chromogenic substrate (SK-4100; Vector). The sections were counterstained with 5× diluted haematoxylin, mounted in 80% glycerol and examined under a light microscope. The information about antibodies used for immunohistochemistry is presented in Supplementary Table S1.

### 4.12. RNA Isolation

Total RNA was isolated from the SCC15CatGFP plus and SCC15CatGFP minus populations using a RNeasy Mini Kit (#74106; Qiagen, Hilden, Germany) according to the manufacturer’s protocol.

### 4.13. RNAseq Analysis

The RNA samples were subjected to quality analysis using a Pico 6000 RNA kit (Agilent, Santa Clara, CA, USA). Based on the measurements, the samples were normalized to a maximum of 50 ng in 10 μL and were subjected to total RNA libraries construction using the KAPA RNA HyperPrep Kit with RiboErase (HMR) (Kapa Biosciences, Oslo, Norway) and KAPA Dual-Indexed adapters (Kapa Biosciences). The manufacturer’s recommendations were used, including fragmentation by incubation 4 min at 94 °C and 14 cycles of enrichment by amplification. The resulting RNA-seq libraries were subjected to fragment length control with an Agilent Bioanalyzer 2100 analyzer and a High Sensitivity DNA kit reagent kit (Agilent, Santa Clara, CA, USA). The library concentration was determined by qPCR using a Kapa Library Quantification kit (Kapa Biosciences). All the procedures were performed according to the manufacturer’s recommendations. Sequencing was performed on the Illumina NovaSeq 6000 instrument using NovaSeq 6000 S1 Reagent Kit (200 cycles) reagents (Illumina, San Diego, CA, USA), in pair-end read mode (2 × 100 cycles) using the standard procedure recommended by producers. The resulting reads were processed as in [74]. Briefly, the reads were trimmed, and Illumina adapters were removed using trimmomatic v0.36 [75] using the following options: LEADING:20 TRAILING:20 SLIDINGWINDOW:6:20 MINLEN:75 CROP:100. The quality of individual fastq files was assessed by FastQC. Subsequently, the remaining rRNAs were removed using sortmeRNA v3.03 [76] and then mapped to hg38 reference transcriptome (GRCh38 UCSC) [77] using the STAR aligner [78]. Due to the contamination of the samples with mouse transcripts and after careful PCA, 1 control sample was removed from the analysis. The aligned reads were then quantified using Salmon v0.13.1 [79] using the following options: validateMappings, rangeFactorizationBins 4, seqBias, gcBias, and numBootstraps 100. Differentially expressed genes (DEGs) were then identified using DESeq2 [80]. Only genes with at least 0.3 log2-fold change and *p*-value less than 0.05 were considered. The obtained results were bioinformatically analyzed through BioVenn [81] and ingenuity pathway analysis (QIAGEN Inc., https://www.qiagenbioinformatics.com/products/ingenuity-pathway-analysis, access date: 6 November 2019) [82].

### 4.14. Statistical Analysis

Statistical significance was determined using student *t*-test. *** *p* < 0.001 was considered to be statistically significant.

## 5. Conclusions

In conclusion, our results demonstrated that using a novel catulin promoter-based reporter system, we can label with GFP and isolate and characterize the specific population of invasive cancer cells enriched in the clusters at the tumor–stroma interface, supporting the collective migration mode of invasion in HNSCC. Those cells harbored the aggressive phenotype as verified by a series of in vitro tests. More importantly, the molecular profiling of those cells provided a list of genes associated with cells movement and invasion. This profile overlapped partially with the expression of signature genes related to the partial EMT available from the single-cell analysis of human HNSCC specimens, highlighting the relevance of our data to the clinical disease progression state. Interestingly, we also observed upregulations of genes involved in axonal guidance like L1CAM, NRP1, semaphorins, and ephrins, emphasizing potential interactions of cancer cells and neuronal components of the stroma.

## Data Availability

Data available on request due to privacy restrictions. The data regarding manuscript are presented in main figures of the article. Additional data obtained in this study are available on request from the corresponding author. The data are not publicly available due to continuation of the project and preparation of next publication.

## Supplementary Materials

The following supporting information can be downloaded at: https://www.mdpi.com/article/10.3390/ijms23010140/s1.

## Abbreviations

Ctnnal1	catenin alpha-like 1
HNSCC	head and neck squamous cell carcinoma
TME	tumor microenvironment
CAF	cancer-associated fibroblast
EMT	epithelial-to-mesenchymal transition
