# Supplementary material for: Catulin Based Reporter System to Track and Characterize the Population of Invasive Cancer Cells in the Head and Neck Squamous Cell Carcinoma
Source: Int J Mol Sci. 2021 Dec 23;23(1):140. doi: 10.3390/ijms23010140 (PMC8745103; doi:10.3390/ijms23010140)
Supplement: Supplementary file 1 [file ijms-23-00140-s001.zip › ijms-1508298-supplementary.pdf]

Supplementary Figure S1

A

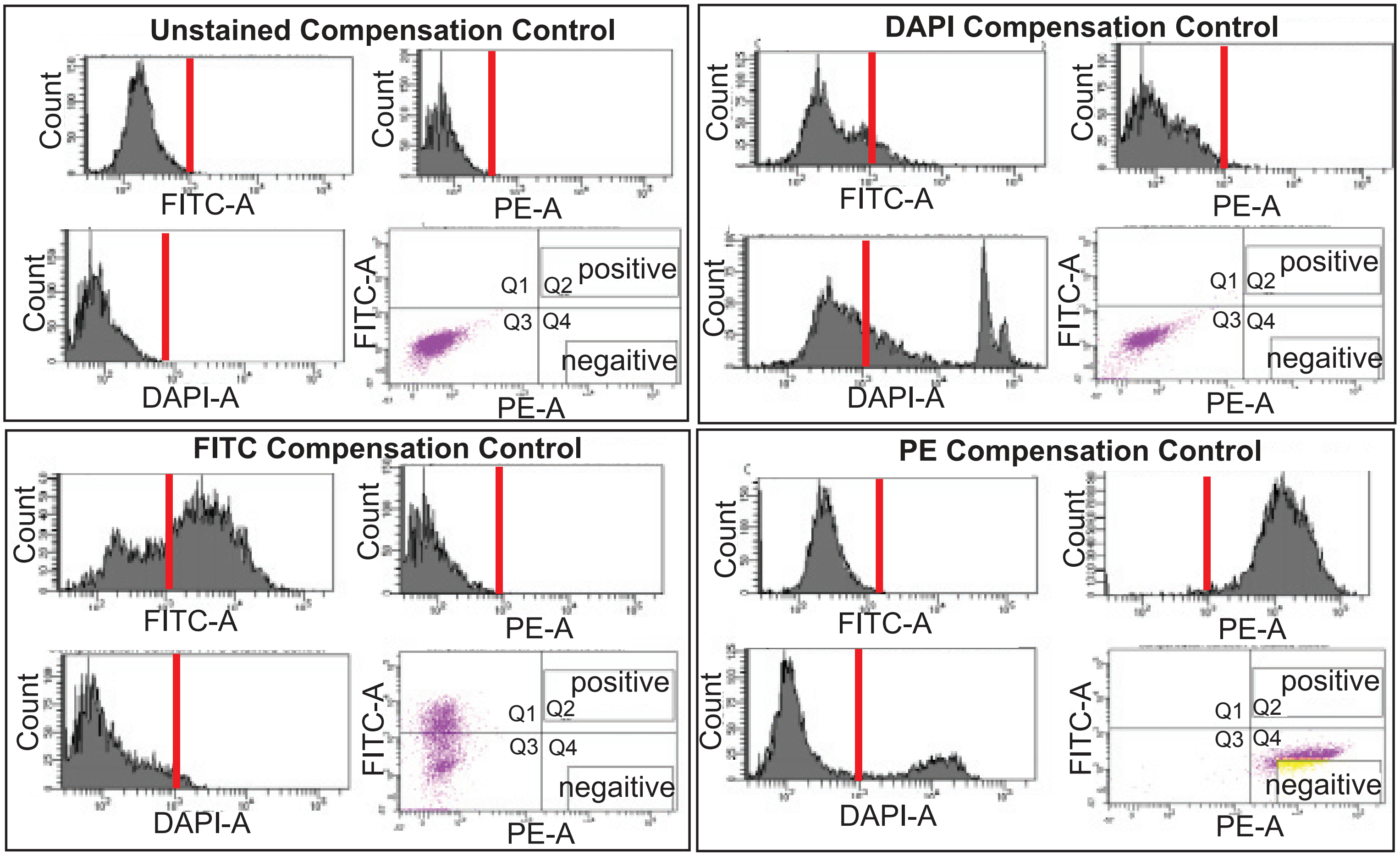

B

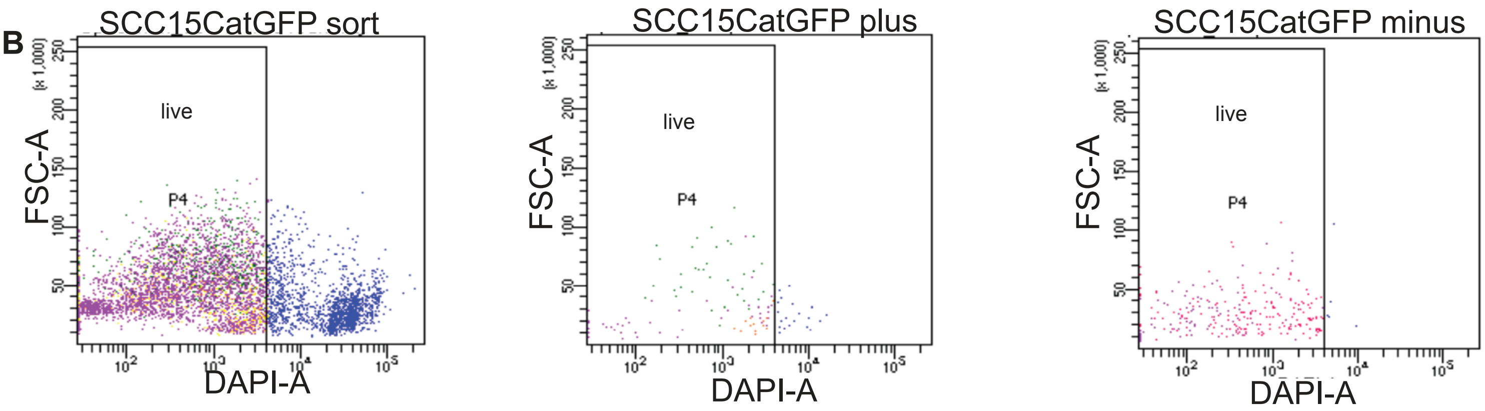

C

	Sort SCC15CatGFP	GFP plus	GFP minus
P4 (DAPI, live)	79	82	98

D

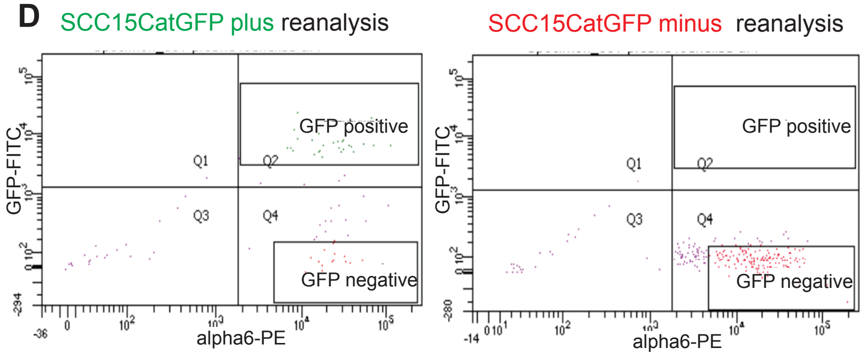

E

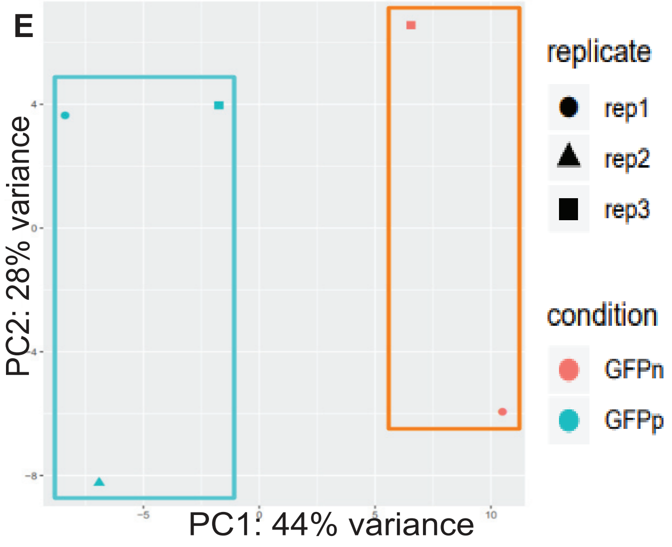

Supplementary Figure S2

A

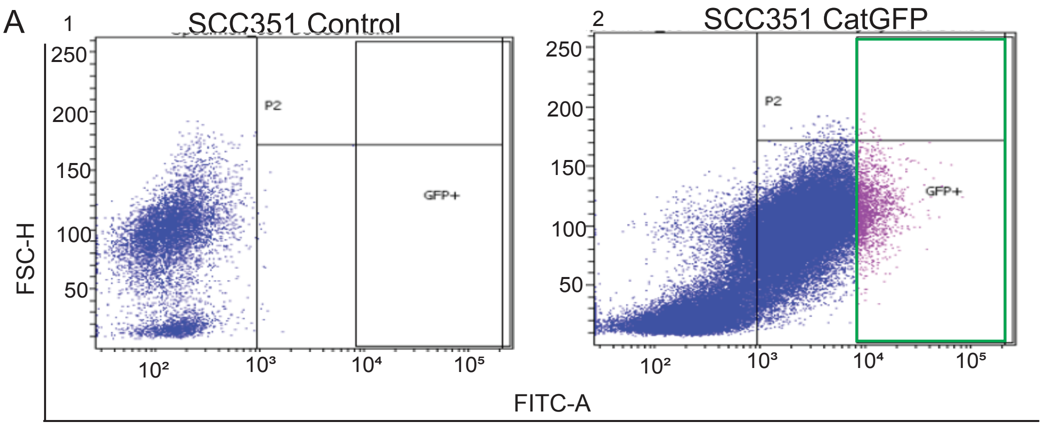

A'

	SCC351 Control	SCC351CatGFP
GFP+	0	1.6
P2	0.3	57.7

B

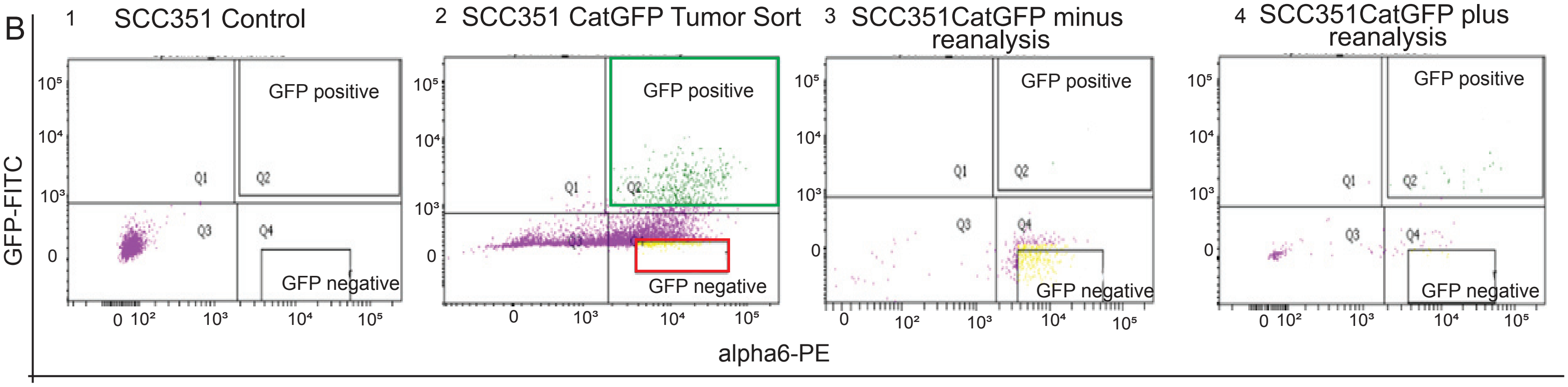

C

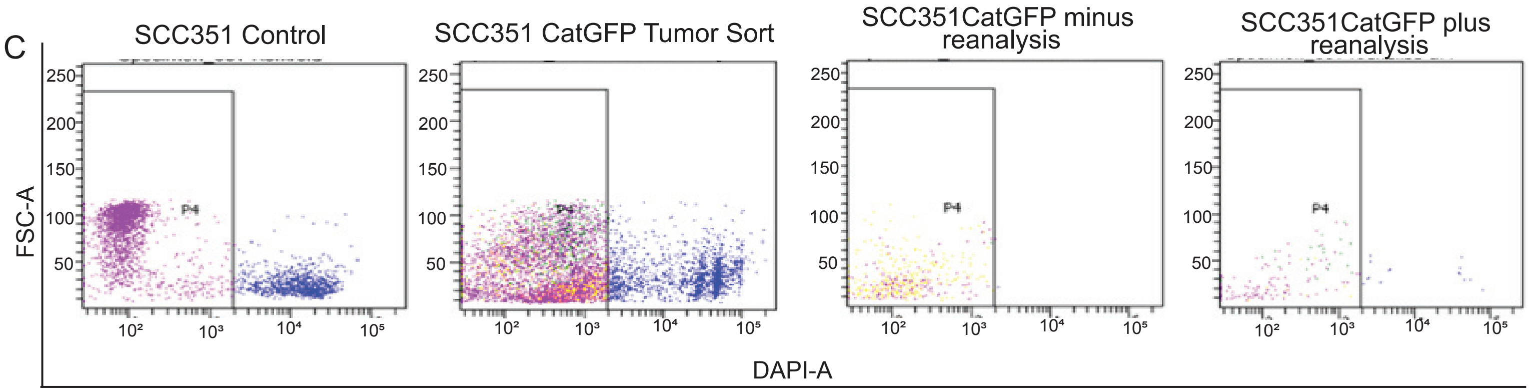

D

	SCC351 Control	SCC351 CatGFP Tumor sort	GFP plus	GFP minus
positive	0	7.8	14.9	0.2
Q2	0	10.5	16.3	0.2
negative	0	10.8	6.4	63
Q4	0	49.6	22	93.6
P4 (live)	71.6	76.2	90.4	99.8

Supplementary Figure S3

A

Categories Increased	Diseases or Function annotation	P-value	Activation Z-score
Cellular Development, cellular growth	Cell proliferation of tumor cell lines	1,42E-25	5,026
Cell cycle	cell cycle progression	2,09E-30	3,292
Cellular Movement	Invasion of cells	2,17E-09	3,277
Cell Death and Survival	Cell survival	3,59E-14	3,026
Cellular Movement	Invasion of tumor cells	9,61E-09	3,017
Cellular Assembly and Organization	Alignment of chromosomes	5,18E-11	2,897
Cellular Assembly and Organization	Formation of filaments	4,30E-08	2,869
Cellular Assembly and Organization	Fibrogenesis	5,32E-08	2,869
Cell cycle, cellular movement	Cytokinesis	1,77E-07	2,844
Cellular Development, cellular growth	Cell proliferation of breast cancer cell lines	2,93E-16	2,826
DNA replication, Recombination and Repair	DNA replication	2,32E-18	2,813
DNA replication, Recombination and Repair	Synthesis if DNA	1,75E-11	2,752
Cell cycle	M phase	1,14E-10	2,693
Cell cycle	Mitosis	1,20E-22	2,679
Cellular Assembly and Organization	Formation of cytoskeleton	3,73E-07	2,659
Cell cycle	Interphase of tumor cells	3,33E-14	2,63
Cell cycle	Interphase	6,85E-23	2,623
Cell cycle	M phase of of tumor cell lines	3,39E-08	2,545
DNA replication, Recombination and Repair	Metabolism of DNA	7,28E-18	2,538
Cell Death and Survival	Cell viability	1,92E-13	2,516
Cell Death and Survival	Cell viability of tumor cell lines	7,00E-15	2,51
Cellular Assembly and Organization	Development od cytoplasm	4,56E-08	2,462
Cell cycle	Interaction of DNA	5,87E-08	2,304
Cell cycle	G1 phase	4,43E-15	2,259
Gastrointestinal Didease	Colorectal disorder	7,30E-21	2,173
Cell cycle	cell cycle progression of cervical cancer cell lines	2,66E-07	2,164
Cancer, Organismal Injury and Abnormalities	Non-hematological solid tumor	6,18E-43	2,017
Cell cycle	G1 phase of tumor cell lines	1,43E-10	2,012

B

Categories Decreased	Diseases or Function annotation	P-value	Activation Z-score
Cellular Assembly and Organization, DNA replication	Formation of gamma H2AX nuclear focus	2,38E-09	-3,732
Cellular Development, cellular growth and proliferation	Hematopoesis of bone marrow cells	1,04E-09	-3,162
Cellular Development, cellular growth and proliferation	Differentiation of hemapoetic cells	5,64E-09	-3,112
Cellular Development, cellular growth and proliferation	Megakaryocytopoesis	8,44E-10	-3,003
Cellular Development, cellular growth and proliferation	Differentiation of hemapoetic progenitor cells	1,43E-08	-2,883
Cell Death and Survival	Necrosis	2,66E-30	-2,85
Cellular Development, cellular growth and proliferation	Hematopoesis of hemapoetic progenitor cells	1,79E-09	-2,838
Cellular development, Hematopoesis, Tissue development	Differentiation of bone marrow cells	4,94E-08	-2,797
Cell Death and Survival	Apoptosis	1,63E-24	-2,737
Cell Death and Survival	Cell death of tumor cell lines	8,95E-33	-2,73
Cellular Development, cellular growth and proliferation	Development of hemapoetic cells	2,56E-08	-2,662
Cellular Development, cellular growth and proliferation	Myelopoesis of hemapoetic progenitor cells	3,07E-09	-2,661
Cellular Assembly and Organization, DNA replication	Formation of nuclear foci	1,09E-08	-2,581
Cellular Development, cellular growth and proliferation	Development of hemapoetic progenitor cells	3,19E-08	-2,526
Hematological System development and function	Development of bone marrow	2,76E-09	-2,501
Cellular Development, cellular growth and proliferation	Development of bone marrow cells	7,92E-09	-2,501
Cell Death and Survival	Cell death of cervical cancer cell lines	7,37E-15	-2,309
Hematological System development and function	Development of hematopoietic system	1,83E-08	-2,122
Cellular Development	Differentiation of tumor cell lines	2,84E-08	-2,023

Suppelmentary Figure S4

A

Invasion of cells only			
UP		DOWN	
CST6	F3	UBE2D3	TNFSF10
CGB8	TNC	ATP6AP1	F2R
FOXC2	PTTG1	CAT	DSG3
LCK	BRCA1	SLC12A6	HTRA1
NES	LOXL2	TFAP2A	SLC48A1
FGFR4	FAS	AHR	HIPK2
SEMA7A	RFC4	CUL7	LTB4R2
FOSL1	EFEMP1	LGMN	MIR31HG
BCYRN1	VAV2	FGFR2	AGR2
CDH2	FHOD1	ZNF652	CEBPA
ETV4	TAGLN2	NCOA2	TGFBR3
IL32	ECT2	SMAD4	NEO1
RRM2	PLAUR	ITGB8	H19
SPHK1	NME1	GRB7	DOCK4
AURKA	CTNNAL1	NOTCH1	SFTPD
SLC7A11	LYN	TOB1	NAALADL2
LOX	FXYD5	LGR4	SATB1
EIF4EBP1	IER3	CTSD	MERTK
NRG1	FABP5	ERBB3	SULF1
DIAPH3	ETS1	FST	SPARCL1
CDK1	STMN1	PDCD4	IGFBP2
KIF2C	FMNL3	MAGI1	CD74
BIRC5	MCAM	ELF3	KCNMA1
TGFA	DIAPH1	UNC5B	ID2
LGALS1	CIB1	SCN8A	CLCA2
FOXM1	PKM		TNS1
AXL	S100A10		
TGFBI	CAP1		
CDKN3	ANXA2		
FBLIM1			

Axonal guidance only	
UP	DOWN
ABLIM3	ITGA2
MYL7	NCK1
KCNJ12	EPHA3
ADAM19	NTF4
ADAMTS16	NTN4
SEMA6B	MMP11
RTN4R	GNG7
GNG4	NFATC1
BMP1	PLXNC1
GNA15	LNPEP
ARPC4	
ARPC5L	
CFL1	

Glioblastoma signaling only	
UP	DOWN
E2F7	FOXO1
RHOF	WNT6
E2F1	RHOV
NF2	
E2F8	
E2F2	

Integrin signaling only	
UP	DOWN
PDGFB	ITGA2
ARPC5L	ARPC4
	ARHGAP5
	NCK1

ILK signaling only
UP
FERMT2
CFL1
KRT18
RPS6KA4
PPP1R14B
PPP2CB

B

Degradation of the extracellular matrix		
GENE	log2FC	pValue
ADAM12	1,606	6,73E-19
ADAM19	1,220	4,96E-06
ADAMTS16	0,931	0,004
ADAM9	0,529	0,006
ADAM15	0,345	0,048

Supplementary Figure S5

A

X- SCC15 and SCCC351 common genes

Y-Invasion of cells
XY TOTAL OVERLAP
CST6
LCK
NES
SEMA7A
FOSL1
IL32
RRM2
SPHK1
AURKA
EIF4EBP1
HMOX1
L1CAM
NRP2
CDK1
KIF2C
BIRC5
TGFA
BCAR3
LGALS1
FOXM1
AXL
CDKN3
SERPINE1
TNC
FAS
RFC4
NRP1
EPHA2
CDK2
TAGLN2
CCND1
ECT2
CTNNAL1
CDK5R1
FXYD5
IER3
EPHB2
PPIF
STMN1
CIB1
PKM
CAV1

Z- Axonal guidance signaling	
XZ TOTAL OVERLAP	XYZ OVERLAP
ABLIM3	L1CAM
ADAM19	NRP2
SEMA6B	NRP1
PDGFB	EPHA2
RTN4R	EPHB2
L1CAM	RHOD
NRP2	
NRP1	
EPHA2	
EPHB2	
ARPC5L	
RHOD	

Z-Glioblastoma multiforme signaling	
XZ TOTAL OVERLAP	XYZ OVERLAP
PDGFB	CDK2
RHOF	CCND1
E2F7	RHOD
CDK2	
CCND1	
RHOD	

Z-Integrin signaling	
XZ TOTAL OVERLAP	XYZ OVERLAP
PDGFB	BCAR3
RHOF	CAV1
BCAR3	RHOD
CAV1	
ARPC5L	
RHOD	

Z-ILK signaling	
XZ TOTAL OVERLAP	XYZ OVERLAP
RHOF	CCND1
CCND1	RHOD
KRT18	
RHOD	

B

Unfavourable prognosis genes	log2FC	pValue	Invasion of cells	Axonal guidance	Glioblastoma multiforme	ILK signaling	Integrin signaling	scc15 &scc351 common
IER3	0,436	0,026						
SERPINE1	0,582	0,054						
PKM	0,325	0,028						
FOSL1	1,127	0,001						
S100A10	0,324	0,024						
BCAR3	0,639	0,014						
CDK2	0,478	0,002						
CCND1	0,464	0,003						
PXN	0,353	0,012						
L1CAM	0,704	0,017						
RTN4R	0,705	0,031						
ACTN1	0,409	0,013						

Supplementary Table S1

List of antibodies used for indirect immunofluorescence and immunohistochemistry.

Antibody	Dilution	Company
E-cadherin	1:100	Zymed Laboratories #13-1900
L1CAM	5ug/ml	Abcam #ab24345
Tenascin C	1:200	Abcam # ab88728
Caveolin-1	1:300	Abcam #ab192869

Supplementary Materials and Methods

Catulin plasmid information

Clone Information: Catalog No.: HPRM14050-PF02

Gene Accession :NM_003798

Description: Homo sapiens catenin (cadherin-associated protein), alpha-like 1 (CTNNAL1)

Promoter Length: 1470 bp

Sequence length upstream of TSS: 1428 bp

Sequence length downstream of TSS: 41 bp

Vector: pEZX-PF02 Vector Size: 4433 bp (backbone only, Promoter insert not counted)

Antibiotic: Kanamycin Stable Selection Marker : Puromycin

Reporter Genes: eGFP Tracking Gene : N/A*

Catulin Primers:

Forward: 5'-AGTTACTTAAGCTCGGGCCC-3'

Reverse: 5'-CCGGACACGCTGAACTTGT-3'

Supplementary Figure S1. A. Compensation controls for FACS sorting: unstained, DAPI, FITC and PE. **B.** Fluorescence analysis for DAPI staining (living cells, viability) of used for sorting SCC15CatGFP cell line and sorted SCC15CatGFP plus and SCC15CatGFP minus population. **C.** Percentage quantification of living cells (p4 gate) in used for sorting SCC15CatGFP cell line (79%) and sorted SCC15CatGFP plus (82%) and SCC15CatGFP minus (98%) population. **D.** Re-analysis of sorted SCC15CatGFP plus and SCC15CatGFP minus population. **E.** Principal component analysis (PCA) of RNAseq analysis for SCC15CatGFP plus (GFPp) and SCC15CatGFP minus (GFPn) sorted populations for three analyzed biological replicates (tumors) (rep1, rep2 and rep3).

Supplementary Figure S2. A. FACS analysis of SCC351CatGFP cells *in vitro.* Fluorescence analysis of control SCC351 cells (1) and reporter cell line SCC351CatGFP (2). **A'.** Percentage quantification of GFP fluorescence in control SCC351 cells and reporter cell line SCC351CatGFP. **B.** Fluorescence analysis of control SCC351 cells (1), sort of SCC351CatGFP (2) cells isolated from formed tumor. Alpha6-PE was used as an epithelial marker. Cells were FACS-sorted into two alpha6-PE- positive population: SCC351CatGFP plus (GFP positive, cells with expression of alpha-catulin, green frame) and SCC351CatGFP minus (GFP negative, cells with no expression of alpha-catulin, red frame). Re-analysis of sorted SCC351CatGFP reporter cell line (SCC351CatGFP minus (3) and SCC351CatGFP plus (4) population. **C.** Fluorescence analysis for DAPI staining (living cells, viability) of SCC351CatGFP control cell line and used for sorting SCC351CatGFP cell line as well as sorted SCC351CatGFP plus and SCC351CatGFP minus population. **D.** Percentage quantification of GFP fluorescence and living cells (DAPI negative, p4 gate) of SCC351 control cells, SCC351CatGFP reporter cell line and sorted SCC351CatGFP plus and SCC351GFP minus population.

Supplementary Figure S3. A. Table of processes (categories) mostly increased in sorted SCC15CatGFP plus population with annotation of specific function. Each color indicate one category (Blue- Cell cycle, green- Cellular movement, pink- Cell death and survival, yellow- Cellular assembly and organization). Invasion of cells is boxed and in bold. **B.** Table of processes (categories) mostly decreased in sorted SCC15CatGFP plus population with annotation of specific function. Each color indicate one category (Yellow-Cellular assembly and organization, purple- Cellular development and Cellular growth and proliferation, pink- Cell death and survival).

Supplementary Figure S4. A. Tables presenting upregulated (in red) and downregulated (in green) genes in SCC15CatGFP plus population that belong only to invasion of cells, axonal guidance, glioblastoma multiforme, Integrin and ILK signaling. Alpha-catulin (CTNNAL1) and TenascinC genes are boxed. **B.** Table presenting genes upregulated in SCC15CatGFP plus population that are associated with degradation of the extracellular matrix.
Supplementary Figure 5. Genes involved in invasion of cells, axonal guidance, Glioblastoma multiforme signaling, Integrin and ILK signaling upregulated in sorted SCC15CatGFP plus population are also upregulated in SCC351CatGFP plus population**. A.** Table presenting upregulated common for SCC15CatGFP plus and SCC351CatGFP plus population genes involved in Invasion of cells, Glioblastoma multiforme signaling, Integrin and ILK signaling. Alpha-catulin (CTNNAL1) gene is boxed. **B.** Table presenting upregulated in SCC15CatGFP plus population genes involved in Invasion of cells, Glioblastoma multiforme signaling, Integrin and ILK signaling that are known as unfavorable prognosis genes. In violet are colored genes that are upregulated in both SCC15CatGFP plus and SCC351CatGFP plus cell lines.

Supplementary Table S1: Information about antibodies used for immunofluorescence and catulin-reporter plasmid information.
